# A serotonergic axon-cilium synapse drives nuclear signaling to alter chromatin accessibility

**DOI:** 10.1016/j.cell.2022.07.026

**Published:** 2022-09-01

**Authors:** Shu-Hsien Sheu, Srigokul Upadhyayula, Vincent Dupuy, Song Pang, Fei Deng, Jinxia Wan, Deepika Walpita, H. Amalia Pasolli, Justin Houser, Silvia Sanchez-Martinez, Sebastian E. Brauchi, Sambashiva Banala, Melanie Freeman, C. Shan Xu, Tom Kirchhausen, Harald F. Hess, Luke Lavis, Yulong Li, Séverine Chaumont-Dubel, David E. Clapham

**Affiliations:** 1 Janelia Research Campus, Howard Hughes Medical Institute, Ashburn, VA, USA; 2 Harvard Medical School, Boston, MA, USA; 3 Boston Children’s Hospital, Department of Pathology, Boston, MA, USA; 4 Howard Huges Medical Institute, Boston Children’s Hospital, Department of Cardiology, Boston, MA, USA; 5 Advanced Bioimaging Center, University of California at Berkeley, Berkeley, CA, USA; 6 Department of Molecular and Cell Biology, University of California at Berkeley, Berkeley, CA, USA; 7 Institut de Génomique Fonctionnelle, Université de Montpellier, CNRS, INSERM, Montpellier, France; 8 School of Life Sciences, Peking University, Beijing, China; 9 Program in Cellular and Molecular Medicine, Boston Children’s Hospital, Boston, MA, USA; 10 Department of Physiology, Faculty of Medicine, Universidad Austral de Chile, Valdivia, Chile; 11 Millennium Nucleus of Ion Channel-Associated Diseases (MiNICAD), Valdivia, Chile; 12 Department of Cell Biology, Harvard Medical School, 200 Longwood Ave, Boston, MA, USA; 13 Department of Pediatrics, Harvard Medical School, 200 Longwood Ave, Boston, MA, USA; 14 Chan Zuckerberg Biohub, San Francisco, CA, USA; 15 Present address: Electron Microscopy Resource Center, Rockefeller University, New York, NY, USA; 16 Present address: Department of Biomedical Engineering, The University of Texas at Austin, Austin, TX, USA; 17 Present address: The University of Wyoming, Laramie, WY, USA; 18 Present address: 10x Genomics, San Francisco, CA, USA; 19 Present address: Yale School of Medicine, New Haven, CT, USA; 20 Present address: Department of Cellular and Molecular Physiology, Yale School of Medicine, New Haven, CT, USA; 21 Lead contact

## Abstract

Chemical synapses between axons and dendrites mediate neuronal intercellular communication. Here, we describe a synapse between axons and primary cilia: the axo-ciliary synapse. Using enhanced focused ion beam-scanning electron microscopy on samples with optimally preserved ultrastructure, we discovered synapses between brainstem serotonergic axons and the primary cilia of hippocampal CA1 pyramidal neurons. Functionally, these cilia are enriched in a ciliary-restricted serotonin receptor, the 5-hydroxytryptamine receptor 6 (5-HTR6). Using a cilia-targeted serotonin sensor, we show that opto- and chemogenetic stimulation of serotonergic axons releases serotonin onto cilia. Ciliary 5-HTR6 stimulation activates a non-canonical G_αq/11_-RhoA pathway, which modulates nuclear actin and increases histone acetylation and chromatin accessibility. Ablation of this pathway reduces chromatin accessibility in CA1 pyramidal neurons. As a signaling apparatus with proximity to the nucleus, axo-ciliary synapses short circuit neurotransmission to alter the postsynaptic neuron’s epigenetic state.

## In brief

Direct engagement of serotonergic axons with cilia extending from pyramidal neurons sets up a serotonin-responsive signaling pathway that can influence chromatin architecture.

## Graphical Abstract

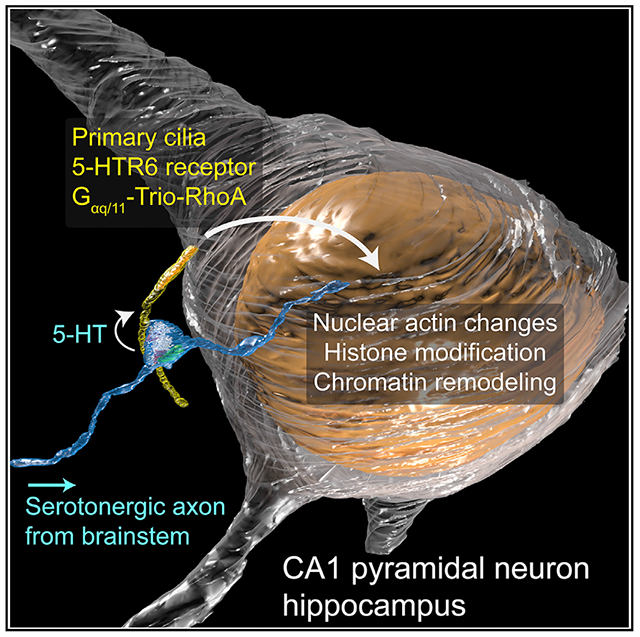

## INTRODUCTION

The primary cilium is a microtubule-based, membrane-bound compartment that extends a few microns from the basal body into the extracellular space (Anvarian et al., 2019). Ciliopathies, genetic disorders caused by mutant proteins related to cilia structure and function, range from embryonic and perinatal death to *situs inversus*, polydactyly, kidney cyst formation, obesity, and neurological deficits. Many of these phenotypes can be attributed to abnormal embryonic development, since the primary cilia house several key components in the Sonic hedgehog (Shh) pathway (reviewed in Goetz and Anderson, 2010).

Less is known about the function of primary cilia in the mature brain in which most neurons no longer divide or differentiate. Although cilia are lost in most terminally differentiated adult skeletal and cardiac muscle, they persist in most mature neurons and glia of the brain (Guemez-Gamboa et al., 2014). Importantly, primary cilia in the adult brain are enriched in certain G-protein-coupled receptors (GPCRs) for neurotransmitters, including dopamine (DA), serotonin (5-HT), and somatostatin (Hilgendorf et al., 2016). Indeed, removal of the ciliary-localized somatostatin receptor 3 (SSTR3) results in novel-object-recognition cognitive impairment without grossly affecting brain development (Einstein et al., 2010). To gain insight into the potential functions of neuronal primary cilia in the adult brain, we set out to determine how ciliary signaling events are activated *in vivo.*

## RESULTS

### CA1 neuronal cilia have a preferred orientation

Anti-adenylyl cyclase 3 (ADCY3) antibodies were used to visualize brain neuronal primary cilia in the hippocampus (Bishop et al., 2007; Figure S1A). The preferred trajectory of hippocampal pyramidal neuron’s primary cilia is along the basal-apical axis (Figure S1B), most strikingly in the CA1 region. Similar preferred orientations of primary cilia were observed in cortical neurons, which aligned with apical dendrites (Kirschen et al., 2017). We next asked if CA1 cilia preferentially project from the deep hippocampus (basal side of pyramidal neurons) to the superficial hippocampus (apical side of pyramidal neurons) to examine a possible morphogen gradient along the basal-apical axis. The base of the cilium was labeled with an antibody against the ciliary rootlet protein, rootletin (CROCC, Yang et al., 2002; Figure S1C). Surprisingly, cilia trajectories were largely bidirectional, with about half of cilia projecting to the more superficial stratum radiatum and the other half projecting to the deeper stratum oriens (Figure S1D). We hypothesized that cilia trajectory is influenced by special contacts between cilia and nearby structures in the neuropil. To test this hypothesis in the mouse brain, we employed volume electron microscopy techniques to visualize neuronal primary cilia and their immediate surroundings.

### FIB-SEM reveals axo-ciliary synapses

**Figure 1.**
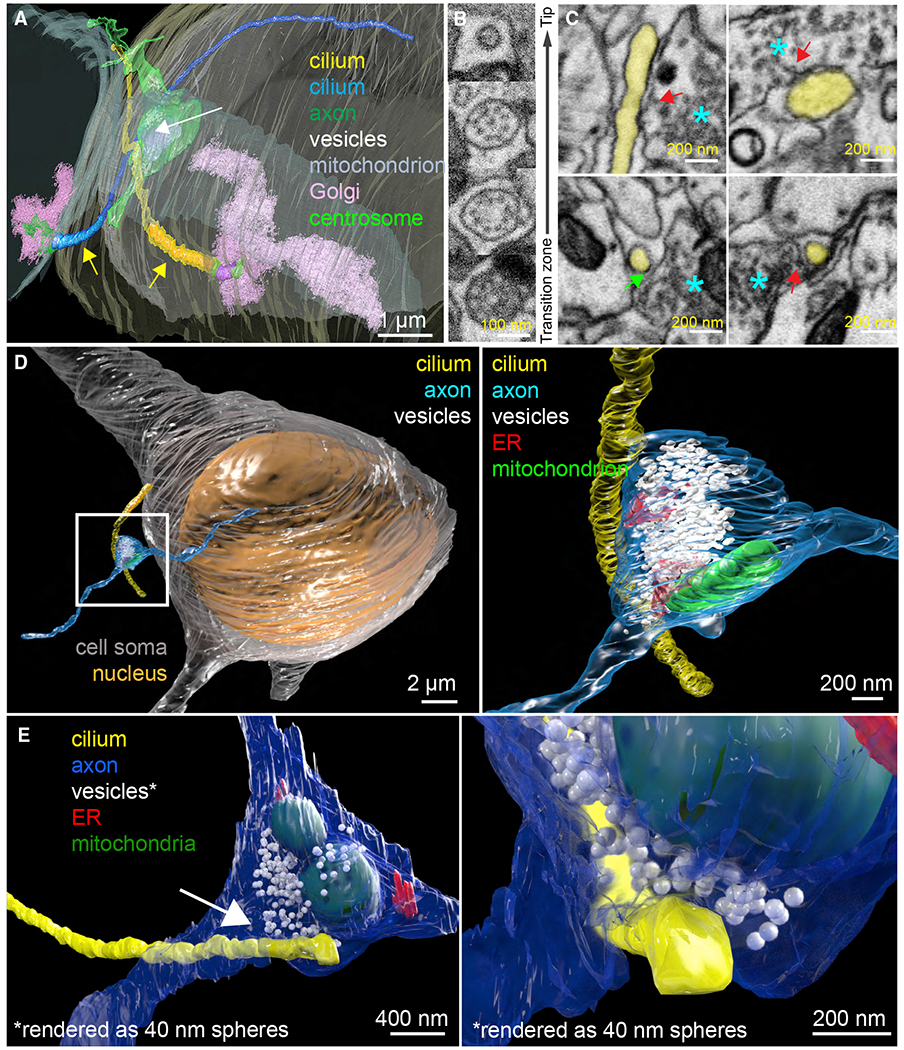
FIB-SEM reveals axo-ciliary synapses (A) Two complete cilia (yellow and blue) arise from the basal bodies (mother centrioles: purple; centrosomes: bright green), which are surrounded by Golgi-related vesicles and Golgi stacks (pink). The axonal varicosity (green) contains a mitochondrion (lavender-gray) and synaptic vesicles (white) and contacts cilia (arrow). Yellow arrows: portions of the cilia that have identifiable microtubule doublets (2–3 μm; colored in saturated yellow and blue, respectively). (B) Primary cilia have a 9+0 microtubule configuration (2nd from bottom) and become 9+1 more distally (2nd from top). No identifiable microtubule doublets are observed in the most distal (6–8 μm) segments (average diameter 100 nm). (C) Single EM sections of axo-ciliary synapses. Cilium: yellow, axon: cyan asterisk. Top left: a reconstructed oblique section showing the longitudinal cross-section of the cilium. In some areas, the cilium and axonal membrane are in direct contact. Occasional vesicles can be seen within 10–20 nm of the axonal membrane opposing the cilium (red arrow). Bottom left: enhanced contrast at the ciliary membrane next to the axon, resembling classic postsynaptic densities (green arrow; cilia-to-axon distance ~20 nm). Top and bottom right: examples suggesting vesicular docking/fusion at the axonal plasma membrane apposing the cilium (red arrows). (D) An axonal process (cyan) gives rise to a varicosity (white box)that makes synaptic contact with a pyramidal neuron primary cilium (yellow; white box magnified at right). (E) A pyramidal neuronal primary cilium (yellow) originates from the left and contacts an axonal varicosity (blue). Area (white arrow) magnified in the right panel. Synaptic vesicles are rendered as 40 nm white spheres to facilitate visualization. Note the axonal ensheathment of the cilium and the axonal vesicle’s proximity to the primary cilium’s membrane. (D and E) Synaptic vesicles: white, endoplasmic reticulum: red, and mitochondrion: green. From 3-month-old C57BL/6J mice.

Focused ion beam-scanning electron microscopy (FIB-SEM) was used to reconstruct the microenvironment of CA1 neuronal primary cilia. In a pilot dataset (6 × 6 × 20 nm^3^ voxel size), we reliably followed two CA1 cilia in a 20 × 20 × 15 μm^3^ volume (Figure 1A). These cilia have variable numbers of microtubule doublets along their length (Figure 1B). The most tantalizing observation was that axonal varicosities often abutted CA1 pyramidal neuronal cilia. In Figure 1A, two cilia meet at an axonal bouton containing synaptic vesicles and a mitochondrion, reminiscent of classical presynaptic axonal terminals. This raised the question of whether pyramidal neuronal cilia are forming specialized contacts with axons and whether these are specialized sites for neurotransmission. We collected 8 FIB-SEM datasets of mature mouse hippocampus at 5.5 × 5.5 × 15 nm^3^ voxel size (30 × 20–30 × 20–30-μm^3^ volumes). We found that most cilia have contact sites with axonal processes (80%, 25/31).

Ciliogenesis of pyramidal neurons starts around birth and continues to elongate, until finally shortening at 8–12 weeks (Arellano et al., 2012). Also, since developing brains exhibit more extracellular space than mature brains (Lehmenkühler et al., 1993), we hypothesized that axo-ciliary synapses might be more evident in younger brains. Indeed, axo-ciliary synapses are evident in FIB-SEM images of P14 mouse brain (Figures S2A and S2B, 83%, 10/12 cilia). In some cases, pyramidal neuronal cilia and axons appear to travel together (Figure S2A). As in adults, mitochondria and the endoplasmic reticulum (with ER-plasma membrane, or ER-PM, junctions) are in axonal processes contacting primary cilia, resembling classic presynaptic boutons (Wu et al., 2017; Figure S2B).

Next, we examined whether fixation artifacts or simply random coincidence were responsible for close contacts. High-pressure freezing-freeze substitution (HPF-FS) of live samples better preserves ultrastructure and extracellular space (Korogod et al., 2015). However, brain samples larger than 10 μm form significant numbers of ice crystals (Korogod et al., 2015). A hybrid protocol of chemical perfusion fixation followed by HPF-FS (Sosinsky et al., 2008) did not preserve the extracellular space and had insufficient contrast for FIB-SEM (not shown). To better preserve ultrastructure and the extracellular space while providing high contrast for FIB-SEM imaging, we optimized perfusion fixation and used imidazole and 3-amino,1,2,4 triazole in osmium-based freeze-substitution staining (Figures S2C and S2D; STAR Methods). We collected two 8-nm isotropic FIB-SEM datasets of adult mouse CA1 samples prepared with the revised hybrid protocol using enhanced FIB-SEM (35 × 35 × 40 μm^3^ and 50 × 50 × 44 μm^3^; Xu et al., 2020). In these two datasets, 18/27 neuronal cilia (67%, Figures 1C–1E; Video S1) contained putative axo-ciliary synapses. 20–40-nm clefts between the axon and the cilium were flanked by areas of immediate membrane apposition between axons and cilia (Figure 1C). In axonal varicosities, vesicles can be seen within 20 nm of the axonal plasma membrane and occasionally appear to be docking or fusing with the plasma membrane, suggestive of vesicular release (Figure 1C, red arrows). Axonal ER-PM junctions and mitochondria in axonal varicosities were again observed (Figures 1D and 1E), along with enhanced contrast at ciliary membranes (Figure 1C, green arrow). These features resemble classical chemical synapses.

### Axo-ciliary synapses are serotonergic

**Figure 2.**
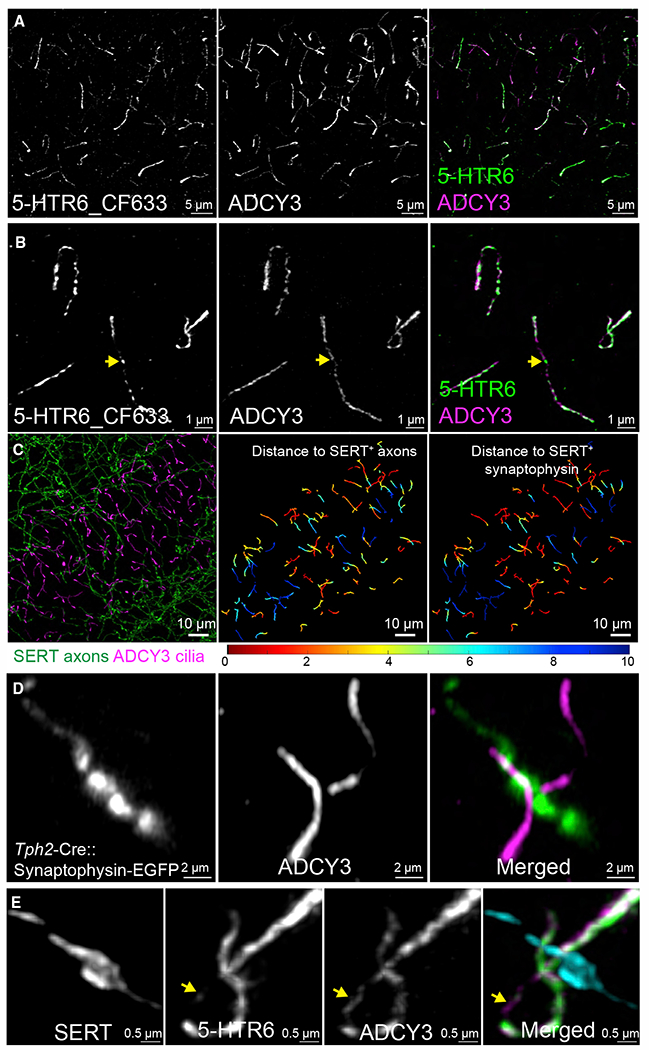
5-HTR6-primary cilia are in contact with serotonergic axonal varicosities (A) HTR6 (labeled by CF633, green in merged panel) is highly enriched in CA1 neuronal primary cilia (ADCY3, magenta in the merged panel: 20 μm MIP). (B) Magnified images from (A). 5-HTR6s are not evenly distributed along the length of cilia and can be enriched at areas with low ADCY3 labeling (arrow). (C) Left panel: cilia (magenta) co-labeled with serotonergic axons (green). 20-μm maximum intensity projection (MIP). Middle panel: cilia in the left panel color coded by the shortest distance to a serotonergic axon. Right panel: cilia from left panel color coded with the shortest distance to a serotonergic axon-associated synaptophysin punctum. (D) Floxed synaptophysin-EGFP driven by *Tph2*-Cre showed ADCY3-labeled cilia (magenta in the merged panel) in contact with serotonergic presynaptic terminals (amplified by anti-GFP and Alexa 488, green in the merged panel). (E) 5-HTR6 (green in the merged panel) are enriched on the cilia at the axonal contact sites (SERT, cyan in the merged panel). Two cilia are in contact with a single serotonergic axonal varicosity. ADCY3 (magenta in the merged panel) can extend beyond the contact site that has few 5-HTR6 (arrow). (A), (B), and (E) are deconvolved confocal images with photon counting detection (Leica). (C) and (D) are Airyscan images (Zeiss). Data from 3- to 4-month-old male C57BL/6J mice.

Since the axons that form axo-ciliary synapses originate from neurons outside the FIB-SEM datasets, we next determined the identity of these axons. 5-hydroxytryptamine receptor 6 (5-HTR6; gene, *Htr6*), a serotoninergic GPCR, is predominantly located in neuronal primary cilia (Brodsky et al., 2017). First, we characterized the location of 5-HTR6 in CA1 pyramidal neurons using an endogenous *Htr6*-EGFP (enhanced green fluorescent protein) knockin mouse line (Nadim et al., 2016). As the expression of endogenous *Htr6* in CA1 pyramidal neurons is low, we amplified EGFP signals with an anti-GFP antibody and a secondary antibody conjugated to a fluorescent dye that has a relatively long fluorescence lifetime (CF633, ~4 ns fluorescence lifetime in phosphate-buffered saline [PBS] and ~2.3 ns in glycerol-based mounting medium as antibody conjugates). This enabled us to use fluorescence lifetimes (FLIM) to separate the 5-HTR6 signals from autofluorescence species (centered around 0.3 ns, Figure S3A; see also Jones et al., 2008), greatly improving the signal-to-background ratio. Co-labeling CA1 pyramidal neuronal cilia with ADCY3 antibody revealed that 94% of the 5-HTR6 signal from the entire cell was ciliary (STAR Methods; Figure 2A). The remaining 6% exists in small puncta, which may be receptors in recycling endosomes or non-specific antibody staining background (Figure S3A). Processing the data with adaptive deconvolution (resolution 120 nm lateral, 200 nm axial) showed that 5-HTR6 receptors are not evenly distributed along the length of cilia but are enriched in segments that are distinct from ADCY3 (Figure 2B).

We labeled pyramidal neuronal cilia with ADCY3 antibody and serotonergic axons with anti-serotonin transporter (SERT, SLC6A4) antibody. This showed that 35% (426/1,209) of cilia are in close apposition to serotonergic axons (Figure 2C; STAR Methods), accounting for ~50% of the axo-ciliary synapses detected in FIB-SEM. In addition, all axonal sites apposing cilia were synaptophysin positive, suggesting that these are serotonin-release sites (Figures 2C and S3B; Belmer et al., 2017). To confirm that the SERT+ synaptophysin puncta arise from serotonergic axons and not from other nearby axons, we co-injected a Cre-dependent synaptophysin-fused EGFP adeno-associated virus (AAV) and tryptophan hydroxylase 2 promoter (Benzekhroufa et al., 2009)-driven-Cre (*Tph2*-Cre) AAV into B8 nuclei of the median raphe, which project to the hippocampus (Muzerelle et al., 2016). Neuronal cilia directly apposed synaptophysin-EGFP puncta or serotonergic presynaptic terminals (Figure 2D). Next, we directly visualized the endogenous 5-HTR6 and serotonergic axons with confocal microscopy with adaptive deconvolution to find that 5-HTR6 was enriched in the subregions of cilia apposing serotonergic axons (Figure 2E).

Since axon-cilia apposition distance could be subject to antibody accessibility and optical chromatic aberrations, we examined the distribution of the shortest distance to the central axes of serotonergic axons (skeletonized axons) among all cilia central axes (skeletonized cilia). The skewed distribution toward short distances (Figure S3C) suggests that ciliary trajectories are biased toward serotonergic axons. Since ligand stimulation is known to result in ciliary remodeling, including ciliary ectocytosis, decapitation, withdrawal, or shedding (Mirvis et al., 2019; Nager et al., 2017), a single snapshot in time may underestimate the frequency of axo-ciliary synapses. Finally, although we found that axon-contacting cilia are slightly longer than non-contacting cilia (Figure S3D; median length 7.0 versus 6.4 μm), we failed to detect any significant correlation between cilia length and the shortest distance to serotonergic axons (Figure S3C), suggesting that simply increasing ciliary length would not result in an increased likelihood of contact with serotonergic axons.

### Activation of serotonergic axons releases serotonin onto cilia

**Figure 3.**
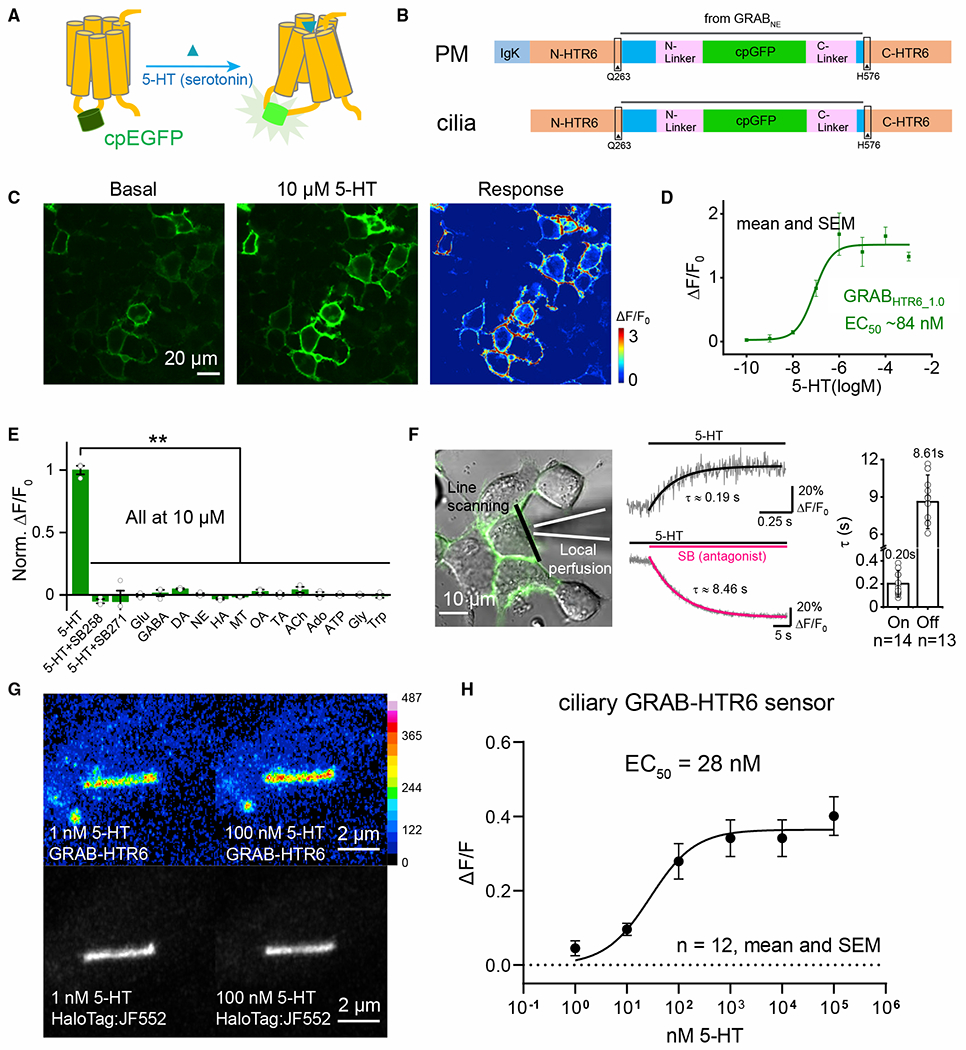
Engineering a 5-HTR6 receptor cilia-targeted sensor (A) A circularly permutated EGFP (cpEGFP) was inserted into the 3rd intracytoplasmic loop of 5-HTR6. Upon ligand binding, a conformational change of the receptor increases EGFP fluorescence. (B) Schematic diagrams of GRAB-HTR6-PM (top) and GRAB-HTR6-cilia (bottom). (C) Representative images show the expression of the GRAB-HTR6-PM sensor (left, no 5-HT; middle, 10 μM 5-HT) and the response (right). (D) Dose-response curve of the GRAB-HTR6-PM sensor. (E) Summary of ΔF/F_0_ measured in GRAB-HTR6-PM-expressing HEK293T cells in response to 10 μM 5-HT, 5-HT with 5-HTR6 antagonist SB 258585 (SB258), or SB 271046 (SB271). ACh, acetylcholine; Ado, adenosine; ATP, adenosine 5′-triphosphate; DA, dopamine; GABA, gamma-aminobutyric acid; Glu, glutamate; Gly, glycine; HA, histamine; MT, melatonin; NE, norepinephrine; OA, octopamine; TA, tyramine;Trp, tryptophan. ΔF/F_0_ was normalized to the averaged peak response measured in 5-HT. Two-tailed Student’s t tests, **p < 0.01. (F) Kinetics of the GRAB-HTR6-PM sensor. Left: a local perfusion system with high-speed line scanning measuring the fluorescence response. Middle: traces of GRAB-HTR6-PM fluorescence in response to 100 μM 5-HT (top) or 100 μM SB 271046 in the continued presence of 1 μM 5-HT (bottom). Right: on- and off-kinetic group data. (G) RPE-1 cells stably expressing a Tet-inducible HTR6-GRAB-cilia-HaloTag. 100-nM application results in increased GFP fluorescence. HaloTag: JF552 was used to reliably identify cilia. (H) Titration curve of the sensor. n = 3 wells, 300–500 cells/well for (D) and (E).

**Figure 4.**
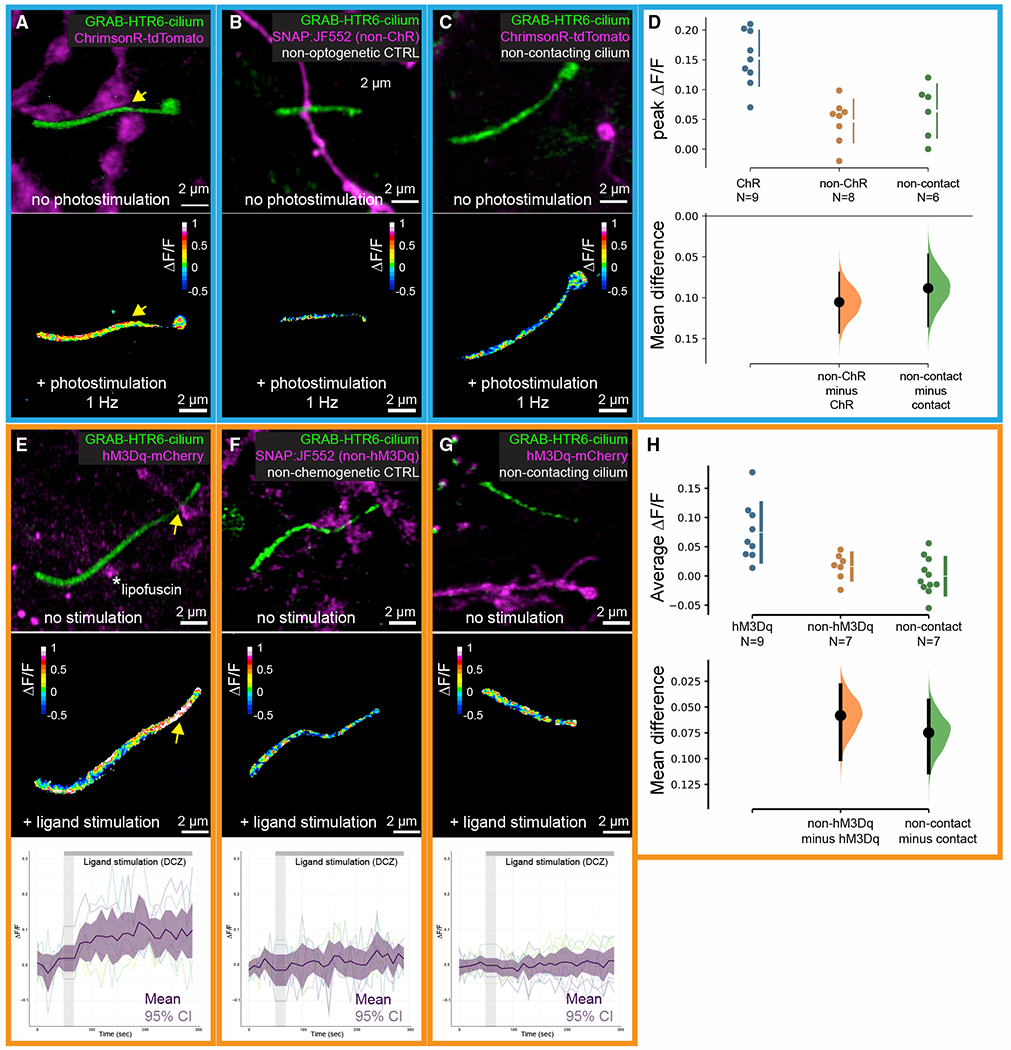
Serotonergic axon activation releases serotonin onto cilia (A–C) Top: a cilium expressing the HTR6-GRAB-cilia sensor in contact with a ChrimsonR-tdTomato-serotonergic axon (ChR, A), in contact with a SNAP:JF552-labeled serotonergic axon (non-ChR control, B), and a cilium distant from a ChrimsonR-tdTomato-serotonergic axon (non-contact, C). Bottom: color maps showing ΔF/F after 25 pulses at 1 Hz optogenetic stimulation, corresponding to the 3 examples in the top row. Arrow in (A) shows the site of contact. (D) Cumming estimation plots of ciliary serotonin levels with optogenetic stimulation of serotonergic axons. ChR mean peak ΔF/F = 0.15. Mean difference between ChR and non-ChR by estimation statistics = −0.10, 95% CI = −0.14 to −0.70, permutation test p value = 0.0002, two-tailed Mann-Whitney test p value = 0.0009. Mean difference between contact and non-contact by estimation statistics = −0.09, 95% CI = −0.14 to −0.05, permutation test p value = 0.005, two-tailed Mann-Whitney test p value = 0.008. (E–G) Top: a cilium expressing the HTR6-GRAB-cilia sensor in contact with an hM3Dq-mCherry-serotonergic axon (E), in contact with a SNAP:JF552-labeled serotonergic axon (nonchemogenetic control, F), and a cilium distant from a hM3Dq-mCherry-serotonergic axon (G). Middle: color maps showing ΔF/F during chemogenetic stimulation by DCZ, corresponding to the 3 examples in the top row. Bottom: individual and averaged traces, corresponding to the 3 examples in the top row. Vertical gray area denotes ligand application. (H) Cumming estimation plots of ciliary serotonin levels with chemogenetic stimulation of serotonergic axons. Average ΔF/F across all time points and samples = 0.08 in hM3Dq. Mean difference between hM3Dq and non-hM3Dq by estimation statistics = −0.06, 95% CI = −0.10 to −0.03, permutation test p value = 0.0076, two-tailed Mann-Whitney test p value = 0.0079. Mean difference between contact and non-contact by estimation statistics = −0.07, 95% CI = −0.11 to −0.04, permutation test p value = 0.0006, two-tailed Mann-Whitney test p value = 0.014. (D and H) Upper: raw data; lower: bootstrap sampling distributions (dot: mean difference; vertical error bars: 95% CI).

To summarize to this point, CA1 pyramidal neuronal cilia receive serotonergic innervation from the raphe nuclei, and ultrastructural analyses provide anatomical evidence of axo-ciliary synapses. However, does activation of serotonergic axons release serotonin onto cilia? To answer this question, we engineered a ciliary-targeted serotonin sensor based on the GPCR-activation-based (GRAB) strategy using the 5-HTR6 receptor as the scaffold (GRAB-HTR6-PM; Figures 3A and 3B; Feng et al., 2019). We first expressed this sensor in HEK293T cells, which trafficked well to membranes with an ~150% fluorescence increase in response to saturating [5-HT] (Figures 3C and 3D; τ_on_ = 0.19 s, τ_off_ = 8.46 s, Figure 3F). The sensor’s EC_50_ to serotonin was 84 nM (Figure 3D; human HTR6 receptor K_D_ = 37 nM; Monsma et al., 1993), with negligible responses to other common neurotransmitters or tryptophan (Figure 3E) and thus could detect ciliary serotonin changes at physiologically relevant levels. To target the sensor to cilia, we removed the exogenous IgK leader sequence in GRAB-HTR6-PM and added a C-terminal HaloTag to better visualize cilia with bright Janelia Fluor dyes (Figure 3G; Grimm et al., 2015; Zheng et al., 2019). This resulted in robust cilia targeting in immortalized human retinal pigment epithelial cells (RPE-1 cells) and neurons, as when HTR6 is expressed (Figure 3G and 4). The EC_50_ for the cilia-targeted HTR6-GRAB-cilia sensor was 28 nM, with up to 40% fluorescence increase per cilium in response to saturating doses of 5-HT (Figure 3H).

We first attempted to image ciliary serotonin dynamics in adult acute hippocampal slices using an AAV sensor construct injected into mice. However, signal-to-noise ratios in deeper areas (>20 μm) were inadequate. Fortunately, hippocampal neuronal primary cilia readily form contacts with serotonergic axons when we co-cultured hippocampal neurons and serotonergic neurons from the raphe nuclei (Figure S4). We expressed the cilia-serotonin sensor GRAB-HTR6-cilia and ChrimsonR, a red-shifted channelrhodopsin (Klapoetke et al., 2014) in hippocampal and serotonergic neurons, respectively. Using Airyscan with 1 Hz photostimulation, we detected serotonin release onto cilia that were in synaptic contact with serotonergic axons (Figures 4A and 4D), an increase that was significantly less in non-channelrhodopsin controls (Figures 4B and 4D). Also, measured serotonin responses were negligible on cilia distant from serotonergic axons (Figures 4C and 4D).

To increase stimulation efficiency, we replaced ChrimsonR in serotonergic neurons with an excitatory muscarinic type 3 receptor DREADD (hM3Dq) (Armbruster et al., 2007; Figure 4E). Application of 10 nM hM3Dq DREADD agonist deschloroclozapine (DCZ, Nagai et al., 2020) reliably increased ciliary serotonin levels with peak ΔF/F up to 0.3 (Figures 4E and 4H). This increase was diminished in non-hM3Dq controls (Figures 4F and 4H). As in the channelrhodopsin experiments, serotonin release was attenuated in cilia distant from serotonergic axons (Figures 4G and 4H). Together, these data suggest that firing serotonergic axons release serotonin onto hippocampal neuronal cilia.

### Serotonin stimulation activates a neuronal ciliary HTR6-G_αq/11_-Trio-RhoA pathway

Plasma-membrane-targeted 5-HTR6 is a G_αs_-coupled GPCR, activating adenylyl cyclase and increasing cAMP in dividing HEK cells (Boess et al., 1997). However, 5-HTR6 activation does not increase ciliary cAMP when it is localized in cultured cell primary cilia (Jiang et al., 2019). GPCR-G protein coupling differs in some ciliated versus non-ciliated cells (Masyuk et al., 2013), and the same GPCR might be coupled to different Gα-subunits on the plasma membrane versus the cilia (Hilgendorf et al., 2019). The G_α11_-subunit (GNA11) was previously identified as a binding partner of the endogenous 5-HTR6 in the brain through affinity purification and mass spectrometry (Nadim et al., 2016). G_αq/11_ can also activate the Trio-RhoA pathway in *C. elegans* and in G_αq/11_-constitutively active mutant uveal melanoma cells (Feng et al., 2014; Williams et al., 2007). Trio is identified in the HTR6-ciliome (Kohli et al., 2017) and is detected in cilia in HTR6-HaloTag RPE-1 cells and in WT-cultured hippocampal neurons (Figure S5A). Indeed, serotonin activates RhoA in HTR6-overexpressing HEK cells and in neurons in which the receptor is distributed throughout the plasma membrane and neuronal processes (Rahman et al., 2017), raising the question of whether HTR6 may signal through the G_αq/11_-Trio-RhoA pathway.

**Figure 5.**
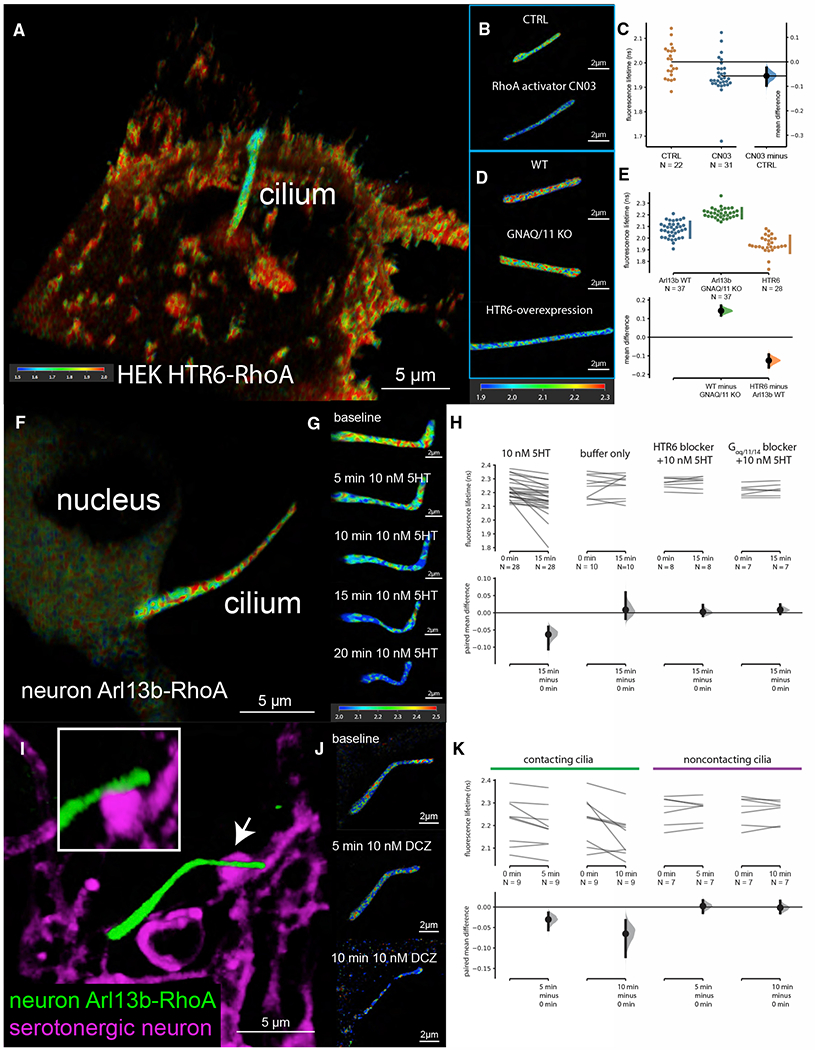
Serotonin stimulation of ciliary HTR6 activates RhoA in cilia (A) HEK293A cells stably expressing the HTR6-RhoA FRET/FLIM sensor. A single cilium arises from the cell soma. (B and C) Cilia-targeted Arl13b-RhoA sensor responds to RhoA activation. Mean difference by estimation statistics = −60 ps, 95% CI = −100 to −24 ps, permutation test p value = 0, two-tailed Mann-Whitney test p value = 0.002. (D and E) GNAQ/11 KO and HTR6-overexpression decreases and increases RhoA activity, respectively. Mean difference between WT and GNAQ/11 KO by estimation statistics = 142 ps, 95% CI = 117–169 ps, permutation test p value = 0, Mann-Whitney test p value < 0.00001. Mean difference between Arl13b and HTR6-cilia by estimation statistics = −125 ps, 95% CI = −162 to −93 ps, permutation test p value = 0, Mann-Whitney test p value < 0.00001. (F–H) 10 nM 5HT stimulation of neuronal cilia increases ciliary RhoA activity. Mean difference at 15 min by estimation statistics = −63 ps, 95% CI = −106 to −41 ps, permutation test p value = 0, Wilcoxon p value = 0.00001. This effect is blocked by either HTR6 blocker SB258585 (100 nM, mean difference at 15 min by estimation statistics = 2 ps, 95% CI = −9–22 ps, permutation test p value = 0.82, Wilcoxon p value = 1) or the G_αq/11_ blocker YM-254890 (1 μM, mean difference at 15 min by estimation statistics = 8 ps, 95% CI = −3–24 ps, permutation test p value = 0.3, Wilcoxon p value = 0.22). Buffer control showed minimal change (mean difference at 15 min by estimation statistics = 8 ps, 95% CI = −17–59 ps, permutation test p value = 0.74, Wilcoxon p value = 1). (I–K) Chemogenetic stimulation of serotonergic axons increases ciliary RhoA activity in contacting cilia (mean difference at 10 min by estimation statistics = −65 ps, 95% CI = −106 to −41 ps, permutation test p value = 0, Wilcoxon rank sum test p value < 0.0001) but not in non-contacting cilia (mean difference at 10 min by estimation statistics = −1 ps, 95% CI = −15–15 ps, permutation test p value = 0.82, Wilcoxon rank sum test p value = 0.81). Arrow in (I) points to area magnified in the inset, which is shown at an oblique angle to demonstrate the close apposition of axon and cilium at the synapse. Contrast is enhanced in the 10 min time point in (J). (C, E, H, and K) Gardner-Altman (C) and Cumming estimation plot (E, H, and K). Upper, raw data; lower, bootstrap sampling distributions (dot: mean difference; vertical error bars: 95% CI).

To measure ciliary RhoA activity, we targeted a FRET-based RhoA sensor (Bindels et al., 2017) to the cilia by fusing it to HTR6 and expressed it in RPE-1 cells (Figure S5C). To better recapitulate the serotonin release at the axo-ciliary synapses, we synthesized a photoactivatable caged serotonin molecule that can be cleaved by 405-nm laser light (Figure S5B). Caged-serotonin stimulation (0.5 Hz) immediately adjacent to the cilium elicited a pulsatile increase in RhoA activity, returning to near baseline upon cessation (Figures S5D and S5E). However, uncaging suffered from a high failure rate, and the FRET ratio could be significantly affected by just a few pixels due to the small size of cilium. To minimize the effect from donor bleaching and better account for the difference in sensor levels, we used FLIM measurements with FRET (FLIM/FRET). As cilia often span multiple Z-levels, we first tested whether FLIM with optical sectioning across the z axis can be achieved using a fast FLIM system equipped with a pulsed white light laser (Harkes et al., 2021). We were able to reconstruct whole HEK293A cells with HTR6-RhoA sensor expression through FLIM imaging (Figure 5A). In this measurement, we expect FRET to decrease the donor lifetime. The Arl13b-RhoA sensor was functional in cilia since stimulation by a RhoA activator (Flatau et al., 1997; Schmidt et al., 1997) decreased the fluorescence lifetime of the donor significantly (Figures 5B and 5C). HTR6-RhoA cilia have higher RhoA activity than Arl13b-RhoA cilia, suggesting that overexpression of HTR6 results in constitutive activity (Figures 5D and 5E), as commonly seen in GPCR signaling (Seifert and Wenzel-Seifert, 2002).

Since cultured hippocampal neurons have ciliary 5-HTR6, serotonin-dependent RhoA activity was then tested in neuronal cilia (Figure 5F). We used a low concentration of 5-HT (10 nM; rat receptor K_D_ ~ 15 nM; Boess et al., 1997) to minimize receptor desensitization and better recapitulate the pulsed nature of serotonin release by axonal firing. 10 nM 5-HT stimulation reliably increased RhoA activity in neuronal cilia in 5–15 min (Figures 5G and 5H). Adding the 5-HTR6 blocker SB258585 (100 nM, Hirst et al., 2000) or a G_αq/11_ inhibitor YM-254890 (1 μM; Nishimura et al., 2010; Takasaki et al., 2004) 5 min before 10-nM 5HT application largely abolished this effect (Figure 5H). In addition, G_αq/11_ knockout (KO) HEK293A cells had significantly lower ciliary RhoA activity (Figures 5D and 5E). Finally, pre-treatment with YM-254890 abolished ciliary RhoA spikes in RPE-1 cells (Figures S5D and S5E). Together, these data suggest that serotonin stimulation results in G_αq/11_-dependent RhoA activation in cilia.

Next, we tested ciliary RhoA activity upon chemogenetic activation of serotonergic axons in the hippocampal neuron-raphe neuron co-culture system. We expressed the Arl13b-RhoA sensor and hM3Dq in hippocampal neurons and raphe neurons, respectively. Application of DREADD agonist DCZ (10 nM) increased RhoA activity in cilia that apposed serotonergic axons within 5 min (Figures 5I–5K). In some cases, we observed ciliary retraction or receptor retrieval from the cilia. In contrast, there was no detectable increase in RhoA activity in non-contacting cilia (Figure 5K). This suggests that the ciliary RhoA activation is under spatial and temporal control of the activity of serotonergic axons.

### 5-HTR6 signaling modulates nuclear actin and histone acetylation

RhoA activation can phosphorylate adducin through Rho-associated kinase and increase its affinity toward F-actin (Fukata et al., 1999). Actin-related lattices in neuronal cell somata revealed by dSTORM imaging assemble on adducin (Han et al., 2017), resembling the classic lattices seen in red blood cells (Bennett and Gilligan, 1993). Consistent with Han et al. (2017), we detected adducin plasma membrane labeling in neuronal cell somata (Figure S6A). When we treated cultured hippocampal neurons with the 5-HTR6 antagonist SB-742457 (Upton et al., 2008), we did not see significant changes in plasma membrane adducin although nuclear adducin was enriched in a small subset of neurons (Figure S6B). This change is reminiscent of nuclear translocation of adducin reported in cultured epithelial cells (Chen et al., 2011; Liu et al., 2017). We next examined adducin staining patterns in the native hippocampal environment. Surprisingly, we did not detect plasma membrane adducin staining in the neuronal cell somata in the hippocampus, but pyramidal neurons exhibited variable numbers of clear nuclear adducin puncta (Figure S6C). In *Htr6* knockout (KO) mouse pyramidal neurons, the density of nuclear adducin puncta increased significantly (Figures S6C and S6D), consistent with our 5-HTR6 antagonist experiments. As *Htr6* transcripts in the hippocampus are not detectable until ~P14 (Thompson et al., 2014), this altered pattern most likely occurs in the late postnatal period or early adulthood. This suggests that ciliary 5-HTR6 signaling is linked to nuclear actin in post-mitotic, post-migration pyramidal neurons.

**Figure 6.**
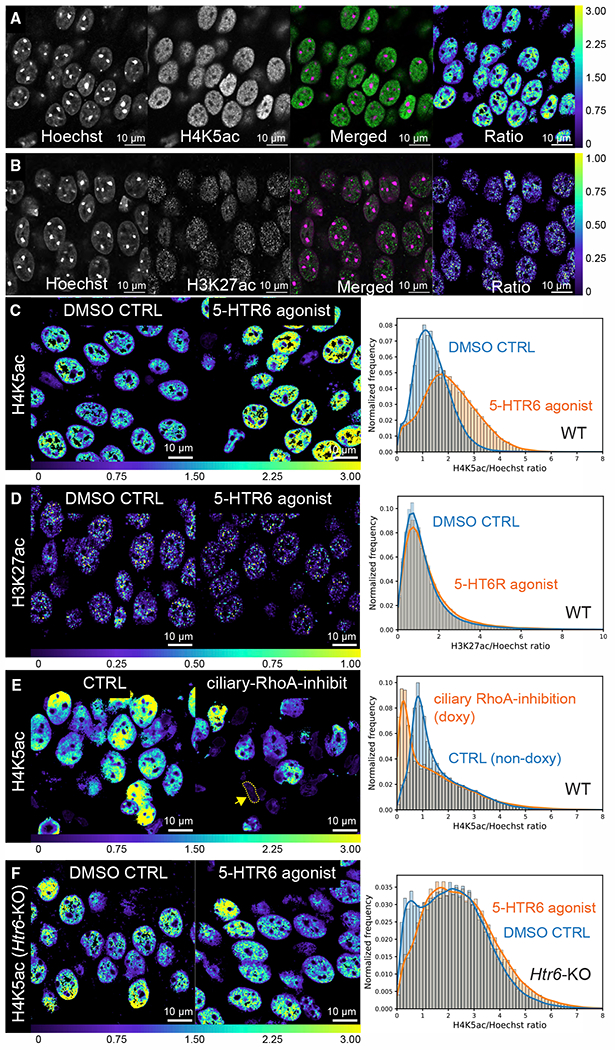
5-HTR6 signaling modulates H4K5 acetylation (A and B) Ratiometric measurements of H4K5ac (A) and H3K27ac levels in fixed mouse brain sections (B). Monoclonal antibodies against H4K5ac and H3K27ac were used to detect histone lysine acetylation (single Airyscan optical sections; green, merged panel). The fluorescent intensity is divided by the Hoechst intensity levels (magenta in the merged panel) to obtain the ratio (rightmost panel, downsampled). (C) 5-HTR6 agonist stimulation increases H4K5ac level. ~60% increase in mode: DMSO: 1.10, WAY181187: 1.76. (D) 5-HTR6 stimulation did not significantly alter H3K27ac level: modes of DMSO and WAY181187 are both 0.77. (E) Ciliary RhoA inhibition for ~1-week decreases H4K5ac level; ~68% decrease in mode: CTRL: 0.82, doxy: 0.26. Many nuclei have small and irregular shapes (arrow). (F) 5-HTR6 agonist stimulation in *Htr6* KO mice does not increase the H4K5ac level. ~24% decrease in mode: DMSO: 2.16, WAY181187: 1.73. (C–F) Left and middle panels: single optical sections of the H4K5ac/Hoechst or H3K27ac/Hoechst ratio. Right panel: histograms with kernel density estimates from entire stacks. Data in (A)–(D) were from 3- to 3.5-month-old and (E) and (F) from 4-month-old male C57BL/6J mice.

Alterations in nuclear actin modify global chromatin (Plessner and Grosse, 2019; Zhao et al., 1998). Nuclear actin directly binds and modulates the activity of histone acetyltransferase KAT14 (cysteine-rich protein 2-binding protein or CSR2B; Viita et al., 2019), a subunit of the histone-modifying human Ada-two-A-containing complex (hATAC). CSR2B significantly acetylates histone H4 lysine 5 (H4K5) *in vitro* and in cells and is modulated by actin monomers (Viita et al., 2019). Therefore, we hypothesized that stimulation of the 5-HTR6 receptor may modulate H4K5 acetylation (H4K5ac) in the hippocampus. Importantly, Park et al. (2013) found H4K5ac to be ubiquitous across the genome and was associated with fear memory. We calculated the H4K5ac to Hoechst ratio on a per voxel basis on Airyscan confocal stacks collected on fixed mouse brain sections, with ~10^6^ voxels after downsampling to isotropic voxels per stack (Figure 6A; STAR Methods). 30 min after injection of the 5-HTR6 agonist WAY-181187 (3 mg/kg, Cole et al., 2007), there was a significant increase in the H4K5ac/Hoechst ratio (Figure 6C). WAY181187 given to *Htr6* KO mice evoked a slight decrease in H4K5ac (Figure 6F), suggesting that the observed increase in H4K5ac is predominantly through 5-HTR6. To further determine whether H4K5ac changes were indeed ciliary RhoA-dependent, we expressed a cilia-targeted TrioRhoGEF inhibitor peptide (Bouquier et al., 2009) under a tetracycline-inducible promoter. In doxycycline-treated adult mice, nuclei were irregular and small with a loss of H4K5ac (Figure 6E), suggesting that ciliary RhoA activity modulates H4K5ac. These changes were also recapitulated using a pan-H4 acetylation antibody (Figure S7). Interestingly, we did not see a significant change in histone H3 lysine 27 acetylation levels (H3K27ac, associated with neuronal activity in the classic Pavlovian contextual fear condition; Marco et al., 2020) with WAY181187 agonist injection (Figures 6B and 6D). This suggests that axo-ciliary signaling may trigger epigenetic programming that is distinct from that of classic neuronal signaling alone.

### 5-HTR6 signaling modulates chromatin accessibility

**Figure 7.**
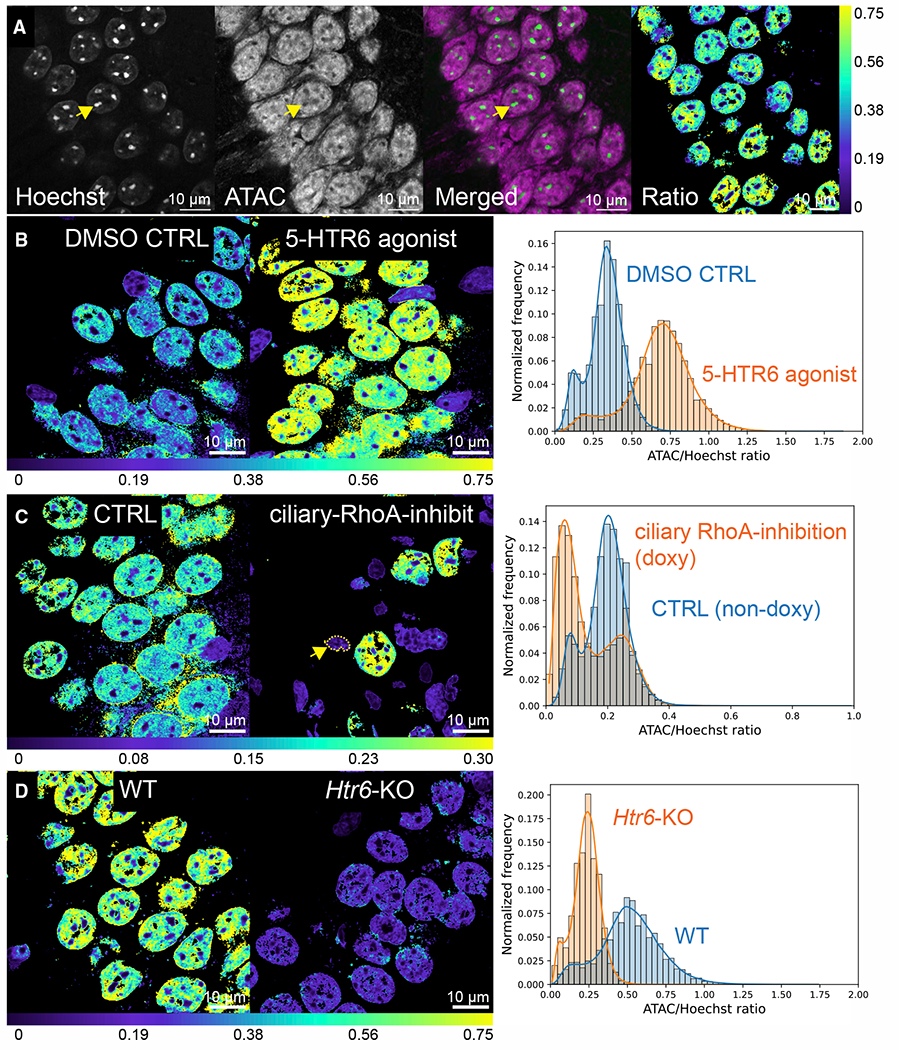
5-HTR6 signaling modulates chromatin accessibility (A) Measurements of chromatin accessibility (ATAC-see) normalized against Hoechst (green) on per voxel bases (right panel). Representative single optical Airyscan section of CA1 pyramidal neurons showing ATAC-see labeling with ATTO-590 dye (magenta). Heterochromatin puncta labeled by Hoechst had little ATAC-see labeling (arrows). (B–D) The ATAC/Hoechst ratio is increased with 5-HTR6 agonist WAY181187 (B, 51% increase in mode: DMSO: 0.34, WAY181187: 0.70), decreased after ciliary RhoA inhibition (C, 70% reduction in mode: CTRL: 0.20, doxy: 0.06), and in *Htr6* KO (D, 52% reduction in mode: CTRL: 0.50, KO: 0.24). Left and middle panels: single optical sections of ATAC/Hoechst ratio. Right: histograms with kernel density estimates from entire stacks. Data in (A) and (B) from 3- to 3.5-month-old and in (C) and (D) from 4-month-old male C57BL/6J mice.

Histone acetylation is associated with increased chromatin accessibility (Marco et al., 2020). ATAC-see is a technique that utilizes a hyperactive transposase mutant with fluorescently labeled oligonucleotides (Chen et al., 2016) to label accessible chromatin in plated monolayers of cells. To apply the technique to tissues, we incorporated the modifications in Omni-ATAC-seq (Corces et al., 2017) and molecular crowding reagents (Picelli et al., 2014) to significantly increase tagmentation efficiency. We were able to achieve reliable ATAC-see labeling in fixed mouse brain sections (Figure 7A). Injection of WAY181187 significantly increased chromatin accessibility as demonstrated by the voxel-based ATAC-to-Hoechst ratio (Figure 7B). Inhibition of ciliary RhoA activity decreased chromatin accessibility (Figure 7C). Chromatin accessibility was also reduced in *Htr6* KO mice (Figure 7D), which may underpin transcription and behavioral changes seen in these mice (Sun et al., 2021). Together, the data suggest that ciliary 5-HTR6 signaling controls chromatin remodeling states via a G_αq/11_-Trio-RhoA pathway.

## DISCUSSION

We presented evidence of synapses between serotonergic axons arising from the raphe nuclei and 5-HTR6 serotonin receptor-expressing primary cilia of CA1 pyramidal neurons. We identified axo-ciliary synapse structures and ciliary 5-HTR6 receptors adjacent to serotonergic axonal varicosities containing vesicles and other markers of synapses. We then demonstrated serotonin release onto cilia upon opto- and chemogenetic stimulation of serotonergic axons. Examining downstream signal transduction in cilia, we provided evidence that ciliary 5-HTR6 can activate the non-canonical G_αq/11_-Trio-RhoA pathway within primary cilia. Finally, we showed that alterations of this pathway in mature neurons change nuclear actin and H4K5ac, thus modulating hippocampal function by altering chromatin accessibility and transcriptional pathways. How these alterations affect the learning and memory deficits seen in *Htr6* KO mice will require a detailed characterization of chromatin accessibility over time with behavioral perturbations and a better understanding of genes and proteins affecting learning and memory circuitry.

The primary finding of the present work is that axons release neurotransmitters onto axo-ciliary synapses and evoke circumscribed signaling to the nucleus that is distinct from signaling at the plasma membrane. Free serotonin levels in the murine hippocampus are ~300 fM (Schechter et al., 2008), while the K_D_ for rat 5-HTR6 binding to serotonin is ~15 nM (Boess et al., 1997). Thus, as is common in neurotransmission, the axo-ciliary synapses localize and concentrate serotonin to achieve a specific function. But unlike traditional synapses, the axo-ciliary synapse may be more analogous to immunological synapses, which feature docking of the mother centriole to the lymphocyte target cell interface while sharing many molecular machineries with the primary cilia (Douanne et al., 2021). Further studies looking more broadly across the central nervous system and with other neurotransmitters will be needed to determine the generality of axo-ciliary synapses.

Not all cilia we examined form serotonergic axo-ciliary synapses. The fact that the portion of cilia with axo-ciliary synapses detected in FIB-SEM (~67%–80%) is greater than those in contact with serotonergic axons (35%) suggests that some CA1 pyramidal neuronal cilia receive different axonal inputs. Indeed, our preliminary data indicated that a different subset of neuronal cilia is in apposition with catecholaminergic axons, which would secrete epinephrine, norepinephrine (NE), or dopamine (DA). This is intriguing since neuronal cilia can also be enriched in other GPCRs, such as the DA receptor 1 (DRD1, Domire et al., 2011), potentially underlying a distinct functional network.

The raphe serotonergic system is much more active in wake states than during sleep (Oikonomou et al., 2019; Wan et al., 2021). HTR6 transcript levels in the brain oscillate (Baldi et al., 2021). A recent preprint suggested that cilia are essential for the rhythmicity of a subset of neurons in the suprachiasmatic nuclei (Tu et al., 2022). We do not know if 5-HTR6 axo-ciliary synapses oscillate and may be involved in chromatin remodeling during sleep/wake cycles (Hor et al., 2019), which may impact learning and memory (Raschand Born, 2013). A recent genome-wide association study identified HTR6 as one of the 15 genes indicated in bipolar disorders (Mullins et al., 2021). Further studies of serotonergic axo-ciliary synapses under physiological contexts may provide insights into neuropsychiatric disorders.

Finally, and most importantly, the ciliary 5-HTR6-Trio-RhoA signaling axis limits serotonin-RhoA signaling to the cilium and exploits its specialized link to the nucleus, much as the Shh pathway regulating Gli transcription factors is limited by compartmentalized G_αs_/PKA signaling. This rationalizes the persistence of primary cilia in non-dividing mature cells such as neurons and can explain how alterations in ciliary signaling can impact structures such as excitatory synapses on dendrites that can be hundreds of microns distant (Tereshko et al., 2021). Interestingly, in other cells such as pre-adipocytes, omega-3 fatty acids were shown to activate primary ciliary FFAR4 receptors to induce CTCF-dependent chromatin changes (Hilgendorf et al., 2019). These findings raise the tantalizing possibility that primary cilia act as an epigenetic regulator to stabilize transcriptional programming in response to environmental cues. In this “cilia as the nuclear antenna” model, cilia provide a protected compartment for shorter, and more direct, encoding of receptor binding to regulate nuclear transcription.

### Limitations of the study

Due to the limited brightness/sensitivity of our cilia-targeted serotonin sensor and the small size of cilia (few sensors /cilium), we were not able to demonstrate serotonin release onto cilia in acute brain slices. Future improvement of the sensor can address this issue. We do not know what initiates the formation and maintenance of axo-ciliary synapses. Preliminary data with light microscopy suggest that serotonergic axons and neuronal primary cilia still form contacts in *Htr6* KO mice, with cilia being 0.5 μm shorter but otherwise well formed. Presumably, adhesion molecules are involved in the establishment of axon-cilium contacts. The 5-HTR6 cilia proteome reveals several adhesion molecules such as L1CAM (Kohli et al., 2017), which is present in the postsynaptic sites of inhibitory synapses (Tai et al., 2019). Further studies on the molecular composition of axo-ciliary synapses may answer these questions and provide molecular handles to perturb these synapses, which could help address some of the limitations of this study. Our work focuses on 5-HTR6 receptor signaling. As a single cilium can have multiple receptors, it will be important to determine how other receptors employ intraciliary signaling molecules, such as calcium and cAMP, to affect their functions. Notably, cAMP/PKA has been shown to decrease RhoA activity by phosphorylation of RhoA or RhoGDIα (Oishi et al., 2012; Qiao et al., 2003). In addition, EPAC, another downstream effector of cAMP, has also been reported to decrease RhoA activity (Yu et al., 2017; Zieba et al., 2011). Careful measurements using cilia-targeted sensors can help determine the integrated output from a single cilium.

## STAR★METHODS

### RESOURCE AVAILABILITY

#### Lead contact

Further information and requests for resources and reagents should be directed to and will be fulfilled by the lead contact, David Clapham (claphamd@hhmi.org).

#### Materials availability

Plasmids generated in this study have been deposited to Addgene: pB-Tet-On-HTR6-RhoA sensor (ID: 189614), pB-Tet-On-Arl13b-RhoA sensor (ID: 189613), pAAV-Syn1-GRAB-HTR6-cilia (ID: 189615), pAAV-TPH2-Cre (ID: 189616), pAAV-TRE-HTR6-SNAPf-TRIP (ID: 189617). Sharing of the RPE-1 cells are limited by terms set by ATCC (American Type Culture Collection). Sharing of HEK239A cell lines are restricted by MTA.

#### Data and code availability

All data reported in this paper will be shared by the lead contact upon request. This paper does not report original code. Any additional information required to reanalyze the data reported in this paper is available from the lead contact upon request.

### EXPERIMENTAL MODEL AND SUBJECT DETAILS

#### Mammalian cell culture

hTERT RPE-1 cells (ATCC CRL-4000, female) and HEK293A cells (female, gift from Dr. Asuka Inoue, Tohoku University, Japan; Schrage et al., 2015) were plated at ~20,000 cells/cm^2^ on #1.5 12 mm coverslips in 24-wells, 1-chamber 35 mm glass-bottom dishes, or 4-chamber 35 mm glass-bottom dishes (all #1.5 cover glass, Cellvis) in 10% serum containing media (RPE-1: DMEM:F12 media, ATCC 30-2006; HEK293A: DMEM, low glucose, GlutaMax, pyruvate, Thermo Fisher Scientific #10567014; Day 0) at 37°C in 5% CO_2_. For HEK293A cells, dishes were coated with Matrigel (Corning Life Sciences) before plating. The next day (day 1), the cells were serum deprived with 0% FBS media with 100 ng/ml doxycycline to induce GRAB-HTR6-cilia, HTR6-RhoA, or Arl13b-RhoA expression. For HEK293A cells, 1 μM H-89 was also added to induce ciliogenesis. After 24 h of serum deprivation (day 2), GRAB-HTR6-cilia cells were labeled with 250 nM Janelia Fluor 552 (JF552) dye for 2 h. Cells were rinsed and placed back in serum-free media without doxycycline or H-89. Experiments were conducted at 48 to 96 h after serum deprivation (day 3 to 5).

For plasma membrane serotonin sensor experiments (GRAB-HTR6-PM), HEK293T cells were cultured in DMEM (Gibco) supplemented with 10% (v/v) FBS (Gibco) and 1% penicillin-streptomycin (Gibco) at 37°C in 5% CO_2_. HEK293T cells were plated on 96-well or 24-well plates and transfected with a mixture of plasmids and PEI (300 ng plasmids and 900 ng PEI for each well in 96-well plates or 1 μg plasmids and 3 μg PEI for each well in 24-well plates) when the cells were grown to ~70% confluence. The medium was replaced after 4-6 h, and cells were used for imaging 24 h after transfection.

For stable cell line creation, RPE-1 cells or HEK293A cells were transfected with the piggyBac hyperactive transposase vector (VectorBuilder) and HTR6-RhoA sensor, Arl13b-RhoA sensor or GRAB-HTR6-cilia vector concurrently with Lipofectomine 3000 (Thermo Fisher Scientific) at a 1:2.5 ratio and grown in 10% Tet-free FBS (Gemini) containing media. The cells were then selected by blasticidin at 10 μg/ml to create Tet-on HT6-RhoA sensor stable cells.

#### Primary hippocampal and raphe neuron culture

Hippocampi and midbrains were dissected from P0 Sprague-Dawley rat pups of both sexes. Rat maintenance and care followed policies advocated by NRC and PHS publications and approved by the Institutional Animal Care and Use Committee (IACUC), Janelia Research Campus. Tissues were digested with papain and gently triturated and filtered through a 40 μm filter. Neurons were electroporated (Lonza 4D-nucleofactor) with Tet-On Arl13b-RhoA (hippocampal neurons) or *Tph2*-Cre (tryptophan hydroxylase 2-Cre, midbrain neurons) and plated in poly-D-lysine coated dishes and cultured in NbActiv4 (BrainBits) at 37°C and 5% CO_2_. A week after plating, the neuronal cultures were fed with B-27 plus neuronal culture system (Thermo Fisher Scientific). AAV transduction (Syn1-GRAB-HTR6-cilia, FLEX-on hM3DGq-DREADD, FLEX-on farnesylated SNAP, or FLEX-on tdTomato) were applied at DIV10. 100 ng/ml doxycycline was added at DIV 6 or DIV14 and removed the next day for Arl13b-RhoA experiments. Images were collected between DIV 9 and DIV 28 (Alr13b-RhoA, hippocampal culture only) or DIV21-35 (hippocampal and midbrain co-culture). JF552-STL (SNAP-labeled neurons) was applied the day prior to imaging (100 nM, 2 h).

#### Animals

Mice used in the study were between P14 (juvenile) and 4-months old (adult; see Figure Legends for the age/sex of each experiment). C57BL/6 and C57BL/6J mice were obtained from Charles River Laboratories and the Jackson Laboratory, respectively. *Htr6*-EGFP knock-in mice were generated at the Institut Clinique de la Souris (Illkirch-Graffenstaden, France). *Htr6* KO mice were generated at the Phenomin consortium (Institut Clinique de la Souris, Illkirch-Graffenstaden, France) by using CRISPR-Cas9. *Htr6* exon 3 and 4 were targeted using two pairs of guide RNAs on each side of the targeted region. Both *Htr6*-EGFP and *Htr6* KO mice were on the C57BL/6 genetic background. All animal work was approved by the Boston Children’s Hospital Institutional Animal Care and Use Committee (IACUC 16-03-3138R), the Janelia Institutional Animal Care and Use Committee (IACUC 16–146 and 19-181), or the animal use and care guidelines of Montpellier University (France, authorization D34-172-4). No restrictions were imposed on food and water. Mice were housed under regular light:dark cycles and standard caging environments. For doxycycline induction experiments, mice were fed with doxycycline-containing food (2000 ppm, Animal Specialties and Provisions, modified from LabDiet 5053) for 1 week. Comparable results were obtained in both males and females (Figures 2, S1, and S3). Littermates of the same sex were randomly assigned to experimental groups.

### METHOD DETAILS

#### Synthesis of PA-O-5-hydroxytryptamine (photoactivatable serotonin)

*N*-Boc serotonin (**S1**, 60 mg, 217 μmol, 3.5 eq) and coumarin bromide (**S2**, 30 mg, 62.2 μmol, 1 eq) were dissolved in CH_3_CN (4 mL). K_2_CO_3_ (potassium carbonate, 60 mg, 435 μmol, 2 eq) was added and the reaction was stirred at room temperature for 15 h. The reaction was concentrated under reduced pressure, the residue dissolved in EtOAc, then washed with water and saturated NaCl (aq), dried over MgSO_4_, and concentrated under reduced pressure. The material was purified using flash chromatography on silica gel (0–50% EtOAc/hexanes, linear gradient), which afforded 35 mg (83%) of compound S3 as a pale-yellow solid. Compound S3 (30 mg, 44.3 μmol) was dissolved in CH_2_Cl_2_ (2 mL). Trifluoroacetic acid (TFA; 0.4 mL) was added and the reaction was stirred at room temperature for 2 h while shielded from light. Toluene (5 mL) was added, and the mixture was concentrated under reduced pressure. The residue was purified by reverse-phase HPLC using a gradient of CH_3_CN/H_2_O containing 0.1% v/v TFA as additive. 1H NMR (400 MHz, 1:1 CD3OD, CD3CN) δ 7.71 (s, 1H), 7.64 (d, J = 8.9 Hz, 1H), 7.31 (d, J = 8.9 Hz, 1H), 7.16 (d, J = 2.4 Hz, 1H), 7.13 (s, 1H), 6.93 (dd, J = 8.8, 2.5 Hz, 1H), 6.65 (dd, J = 9.0, 2.7 Hz, 1H), 6.52 (d, J = 2.6 Hz, 1H), 6.32 (s, 1H), 5.30 (s, 2H), 4.24 (s, 4H), 3.15 (t, J = 7.3 Hz, 2H), 3.01 (t, J = 7.3 Hz, 2H). HRMS (ESI) calculated for C_24_H_24_N_3_O_7_ [M+H]+ 466.1609, was 466.1615.

#### Design and cloning of RhoA and 5-HT sensors

The piggyBac Tet-On HTR6-RhoA sensor was generated based on the published mScarlet-I based RhoA sensor (Bindels et al., 2017). The mScarlet-I:sGFP2:RhoA and the cpPKN1 fragments were synthesized (Genscript) and cloned into piggyBac Tet-On vector Xlone HTR6-HaloTag (Deo et al., 2019). The Xlone-Ar13b-RhoA sensor was subcloned by replacing HTR6 with ARL13B (VectorBuilder).

To make GRAB-HTR6-PM, cpEGFP and linkers were PCR-amplified from GRAB_NE_ (Feng et al., 2019), and the cDNA encoding human 5-HTR6 was PCR-amplified from HTR6-Tango (a gift from Bryan Roth, Addgene 66414; RRID:Addgene_66414; Kroeze et al., 2015). Then, the chimeric GRAB sensor was cloned into the pDisplay vector (Invitrogen). Similar to other sensors based on the same platform, the N-terminus of this construct has an IgK leader sequence that serves as a plasma membrane targeting signal to further enhance the plasma membrane expression (see Methods S1 for full amino acid sequence). In addition, an IRES-mCherry-CAAX was added at the C-terminus to serve as a reference of membrane marker to calibrate the expression levels. GRAB-HTR6-cilia was made by cloning the GRAB-HTR6-PM (excluding the IgK leader sequence and IRES-mCherry-CAAX) with a 3xGGGGS-HaloTag at the C-terminus onto the Xlone backbone by VectorBuilder. The wild-type 5-HTR6 localized well to cilia (Barbeito et al., 2021; Berbari et al., 2008) without the artificial IgK leader sequence. Since the sensor inherits the properties of 5-HTR6, it can still traffic well onto the ciliary membrane.

#### Molecular Biology of AAV vectors

*Tph2*-Cre vector was generated by replacing GFP in *Tph2*-GFP(Wan et al., 2016; gift from Dr. Björklund) with the Cre-recombinase in Syn1-EBFP-Cre AAV (gift from Hongkui Zeng, Addgene plasmid # 51507; RRID:Addgene_51507). pAAV-Syn-FLEX-rc[ChrimsonR-tdTomato] was a gift from Edward Boyden (Addgene plasmid # 62723; RRID:Addgene_62723). pAAV[FLEX-ON]-CAG-Farnesylated-SNAPtag AAV plasmid was made by VectorBuilder, Inc. pAAV-FLEX-ON-tdTomato was a gift from Edward Boyden (Addgene plasmid # 28306; RRID:Addgene_28306). pAAV-EF1a-DIO-hM3D(Gq)-mCherry was a gift from Bryan Roth (Addgene plasmid # 50460; RRID:Addgene_50460). pAAV-TRE-HTR6-SNAPf-TRIP and pAAV-Syn-Tet3G were made by VectorBuilder. Syn-Tet3G and TRE-HTR6-SNAPf-TRIP were packaged in AAV2/rh10 capsid, and the other viral vectors were packaged in the AAV2/PHP.eB capsid. All above viral vectors were made by the HHMI Viral Tools (Janelia). AAV phSyn1(S)-FLEX-tdTomato-T2A-SypEGFP-WPRE was a gift from Hongkui Zeng (Addgene viral prep # 51509-AAV1; http://n2t.net/addgene:51509; RRID:Addgene_51509).

#### Intracranial injections of AAVs

8-week-old adult mice were anesthetized with 2.5%-3.0% isoflurane with an O_2_ rate of 1L/min and mounted on a stereotaxic frame. The body temperature was maintained at 37°C using a heating pad. Mice were given buprenorphine (0.1 mg/kg) subcutaneously at 5 mg/kg of body weight. After shaving, a drill was used to create a small craniotomy hole (~1 mm). For CA1 pyramidal neuron layer injections, 200 nL of AAV mixture composed of AAV-rh10-Syn-Tet3G and AAV-rh10-TRE-HTR6-TRIP (1:2 ratio, 5×10^12^ vg/ml and 10^13^ vg/ml, respectively) were bilaterally injected at anteroposterior −2.25 mm relative to Bregma, mediolateral ±2 mm relative to Bregma; dorsoventral −1.43 mm relative to the skull surface at a rate of 50 nL/minute using a Nanoject II Injector (Drummond Scientific, USA) followed by 5 additional minutes to allow diffusion. For median raphe B8 injections, 750 nL of AAV mixture composed of AAV-php.eB-*Tph2*-Cre and AAV-2/1-phSyn1-FLEX-tdTomato-T2A-SypEGFP (1:2 ratio, 5×10^12^ vg/ml and 10^13^ vg/ml, respectively) were injected at anteroposterior −4.4 mm relative to Bregma, mediolateral 0 mm relative to Bregma; dorsoventral −4.4 mm relative to the skull surface at a rate of 75 nL/minute using a Nanoject II Injector (Drummond Scientific, USA) followed by 5 additional minutes to allow diffusion. Upon recovery, mice were given Ketaprofen (5 mg/kg, subcutaneous) and placed back in the home cage. Ketoprofen (5 mg/kg, sub-cutaneous) was given once a day for additional two days post-surgery.

#### Immunofluorescence and imaging

For cultured cells, #1.5 coverslips were first fixed in 4% paraformaldehyde (PFA) in PBS overnight at 4°C. Samples were then rinsed in PBS for 5 min x 3. After permeabilization with 0.3% Triton-X in PBS for 30 min, samples were blocked with 5% normal goat serum (NGS) in PBS for 1 h. 5% NGS was then replaced with primary antibody containing solution in 1% BSA in PBS with 2 mM sodium azide overnight at 4°C. After rinsing in PBS for 5 min x 3, samples were stained with secondary antibody and Hoescht 33342 for 2 h. For Trio staining, the samples were stained according to the Alexa tyramide amplification system with the SuperBoost protocol (Thermo Fisher Scientific). Samples were then rinsed in PBS for 5 min x 2 /Milli-Q water 5 min x 1 and mounted on Vectashield Vibrance (Vector Laboratories) or Prolong Fade Glass (Thermo Fisher Scientific).

For mouse brain samples, mice were deeply anesthetized with isoflurane inhalation or ketamine/xylazine (200 mg/kg ketamine and 20 mg/kg xylazine) intra-peritoneal injection and intracardially perfused with 4% PFA in phosphate-buffered saline (PBS). The brains were removed and post-fixed in 4% PFA in PBS overnight at 4°C. Samples were then rinsed in PBS x3, 15 min each. Serial sections of 50 μm or 200 μm were obtained on a vibratome. For 50 μm mouse brain sections, fixed slices were incubated at room temperature in 0.3% Triton-X in PBS overnight, followed by 5 h in blocking buffer (BlockAid, Thermo Fisher Scientific), overnight in primary antibody, and again overnight in secondary antibody and Hoechst 33342 (2 μg/ml). Both primary and secondary antibodies were diluted in the same blocking buffer (BlockAid, Thermo Fisher Scientific). The stained sections were then rinsed in PBS 15 x 2 times/300 mOsm glycerol 15 min x 1 and mounted in ProLong Fade Glass or SlowFade Glass (Thermo Fisher Scientific). Between antibodies, slices were washed x3, 15 min each with PBS. For 200 μm mouse brain sections, slices were first treated with the epoxide-crosslinker as in the SHIELD protocol (Life Canvas Technology, Park et al., 2018). Afterwards, sections were incubated in 2% Triton-X in PBS with 2 mM sodium azide for 3 days for permeabilization. Sections were then incubated in primary and secondary antibodies for 2 days at room temperature, respectively. After washing, sections were mounted in SlowFade Glass (Thermo Fisher Scientific).

The following primary antibodies were used: rabbit anti-GFP (Chromotek PABG1, 1:1000), guinea-pig anti-SERT (synaptic systems 340004, 1:200), mouse anti-ADCY3 (Encor Biotechnology MCA-1A12, 1:1000), rabbit anti-PCP4 (Millipore Sigma, HPA005792, 1:500), chicken anti-rootletin (Millipore Sigma, ABN1686, 1:1000), rabbit anti-ADD1 (Abcam EP734Y, #ab40760, 1:250), rabbit anti-synaptophysin (Cell Signaling, #36406, 1:100), rabbit anti-synaptophysin (Thermo Fisher Scientific, MA5-14532, 1:200), rabbit anti-Trio GEFD2 (custom antibody, gift from Dr. Susanne Schmidt, 1:500), rabbit anti-H4K5ac (Thermo Fisher Scientific MA5-32009, 1:250), mouse anti-panH4ac (Thermo Fisher Scientific MA3-066, 1:250), rabbit anti-H3K27ac (Abcam ab177178, 1:7000). The following secondary antibodies and dyes were used: Alexa Fluor Plus 488 goat anti-rabbit (1:1000, Thermo Fisher Scientific), CF488A donkey anti-guinea pig (1:1000, Biotium), Alexa Fluor Plus 555 goat anti-rabbit (1:1000, Thermo Fisher Scientific), CF555 goat anti-mouse IgG_1_ (1:1000, Thermo Fisher Scientific), CF633 goat anti-mouse IgG_1_ (1:1000, Biotium), CF633 donkey anti-rabbit IgG_1_ (1:1000, Biotium), Hoescht 33342 (10 μg/ml, Thermo Fisher Scientific), SuperBoost goat anti-rabbit polyHRP (ready-to-use 1x concentration, Thermo Fisher Scientific).

Confocal imaging was done either on a Zeiss 880/980 Laser Scanning Confocal Microscope (LSM) or Leica Stellaris with FLIM. The Zeiss systems are equipped with a Plan-Apochromat 63x/1.4 oil, 40x/1.2 multi-immersion LD LCI Plan-Apochromat, and 20x/0.8 air Plan-Apochromat objective; ZEN Black and Blue software. Hoechst 3342 was excited by 405 nm laser light and the spectral detector set to 409-481 nm. Alexa 488/CF488 was excited by 488 nm laser light and the spectral detector set to 490-545 nm. Alexa 555 was excited by 561 nm laser light and the spectral detector set to 570-642 nm. ATTO-590 was excited by 594 nm laser light (Airyscan only). CF633 was excited with 633 nm light and the spectral detector set to 642-755 nm. The spectral detector was only used for non-Airyscan confocal scanning imaging sessions. The Leica Stellaris is equipped with a 20x air HC PL APO 20x/0.75 CS2 and HC PL APO 63x/1.4 oil-immersion objectives; Las X software. Alexa 488 was excited at 499 nm and imaged with the HyD X2 detector set to a 504 - 548 nm window. CF555 was excited at 553 nm and imaged with the HyDS3 detector set to a 558 - 633 nm window. CF633 was excited at 629 nm and imaged with the HyDX4 detector set to a 637 - 750 nm window. All detectors were set in photon counting mode, and the pulsed white light laser was set at 80 MHz. With the CF633 channel, the average photon arrival time was used to separate autofluorescence background (peak at 0.3 ns) and CF633 signal (peak at 2.3 ns). Adaptive deconvolution was performed using Leica Lightning processing with smoothing set to “very low” and without cutoff or auto-contrast.

#### Electron microscopy of CA1 pyramidal neuron cilia

##### Conventional chemical fixation protocol

Mice were anesthetized with ketamine/xylazine (200 mg/kg ketamine and 20 mg/kg xylazine) intra-peritoneal injection and perfused with a solution of 2% PFA and 2% glutaraldehyde in 0.1 M sodium cacodylate buffer, 0.2 mM CaCl_2_. The brain samples were dissected and post-fixed in the perfusion solution overnight at 4°C. After rinsing with 0.1M sodium cacodylate buffer, 300 μm serial sections were obtained on a vibratome. The tissue was then immersed in 1% osmium tetroxide and 1.5% potassium ferricyanide in a 0.1 M cacodylate buffer for 1 h. After rinsing in 0.1 M sodium cacodylate buffer, the sections were further stained with 1% osmium tetroxide in water for 1 h, followed by 2% uranyl acetate in maleate buffer (pH = 5.15) overnight at 4°C. The tissue was then washed in water, dehydrated with graded ethanol, and embedded in Epon812 resin.

Epon-812 flat-embedded mouse hippocampal CA1 samples were first mounted on an aluminum stub. The sample surface was polished on an ultramicrotome, followed by carbon coating (20 nm). The samples were then imaged on the Zeiss Crossbeam 540 at 5-6 nm pixel size with 15-20 nm milling using ATLAS 5 software (Zeiss).

##### Hybrid protocol

2 - 3-month-old male C57/BL6 mice were deeply anesthetized and transcardially perfused with 30 mL of 3% PFA (60 mM NaCl, 130 mM glycerol, 10 mM sodium phosphate buffer). The brain was carefully dissected from the skull and post-fixed with 50 mL of 3% PFA (30 mM NaCl, 70 mM glycerol, 30 mM PIPES buffer, 10 mM betaine, 2 mM CaCl_2_, 2 mM MgSO_4_) at room temperature for 2 h. The brain sample was then rinsed in a 400 mOsM buffer (65 mM NaCl, 100 mM glycerol, 30 mM PIPES buffer, 10 mM betaine, 2 mM CaCl_2_, and 2 mM MgSO_4_) for 0.5 h, followed by vibratome sectioning (coronal sections, 100 μm thickness) using a Leica VT1000S vibratome in the same buffer. 100 μm sections were then fixed in 1% PFA, 2% glutaraldehyde solution (30 mM NaCl, 70 mM glycerol, 30 mM PIPES buffer, 10 mM betaine, 2 mM CaCl_2_, 2 mM MgSO_4_, 75 mM sucrose) overnight at 4°C. Sections were then washed using the 400 mOsM rinsing buffer (see above). Round samples of the hippocampus were created from the 100 μm coronal sections using a 2 mm biopsy punch (Miltex). The 2 mm samples were dipped in 1-Hexadecene, placed in a 100 μm aluminum carrier, covered with a flat carrier and high-pressure frozen using a Wohlwend compact high-pressure freezer (Wohlwend GmbH, Switzerland). Samples were then freeze-substituted in 0.5% osmium tetroxide, 20 mM 3-amino-1,2,4-triazole or 20 mM imidazole, 0.1% uranyl acetate, 4% water in acetone, using a Leica AFS2 system. Specimens were further dehydrated in 100% acetone and embedded in Durcupan resin.

Two datasets were acquired using the hybrid protocol, stained with either osmium-imidazole or osmium-3-amino-1,2,4-triazole. The samples were mounted on a copper post and trimmed to the Region of Interest (ROI), guided by X-ray tomography data obtained by a Zeiss Versa XRM-510. The samples were coated with a thin layer of 10-to 20-nm gold and 50-to 100-nm carbon and imaged by a customized Zeiss Merlin FIB-SEM or NVision40 FIB-SEM system using 8 nm pixel size with 2 or 4 nm of milling depth. After alignment using a Scale Invariant Feature Transform (SIFT) based algorithm (Lowe, 2004), the stacks were binned by a factor of 2 or 4 along z to form a final isotropic volume of 35 μm x 35 μm x 40 μm and 50 μm x 50 μm x 44 μm with 8 nm x 8 nm x 8 nm voxels.

The electron microscopy datasets generated above were manually segmented using VAST (Volume Annotation and Segmentation Tool, Berger et al., 2018). Segmented results were exported as obj files, and rendered using 3ds Max 2021 (Autodesk, Inc.). For synaptic vesicles in Figure 2E, the centroids of segmented vesicles were calculated in 3D, and 40 nm spheres were generated as 40 nm spheres in 3ds Max.

The hybrid protocol was developed for this work, which were used to generate two enhanced FIB-SEM datasets in the CA1 area to examine primary cilia. Prior to the completion of this manuscript, these datasets have been shared with others to examine lipid droplets (Ioannou et al., 2019), myelin distribution along large axons (Gao et al., 2019), and mitochondrial morphology (Delgado et al., 2019).

#### Characterization of the GRAB-HTR6-PM sensor

For dose-dependent curve and selectivity tests, HEK293T cells expressing the GRAB-HTR6-PM sensor were imaged by the Opera Phenix high-content screening system. Before imaging, the culture medium was replaced with 100 μL Tyrode’s solution consisting of (in mM): 150 NaCl, 4 KCl, 2 MgCl_2_, 2 CaCl_2_, 10 HEPES and 10 glucose (pH 7.4). For imaging, a 40x/1.1-NA water-immersion objective, a 488-nm laser combined with a 525/50-nm emission filter for excitation and collection of the GFP fluorescence signal, and a 561-nm laser combined with a 600/30-nm emission filter for the collection of the mCherry fluorescence signal were used. The same field of views (FOVs) were imaged without or with 5-HT (at various concentrations, in Tyrode’s solution), respectively. The fluorescence signal of the GRAB-HTR6-PM sensor was calibrated using the ratio of GFP to mCherry.

For kinetics measurement, we used an inverted Ti-E A1 confocal microscope (Nikon) equipped with a 40x/1.35 numerical aperture oil-immersion objective, a 488-nm laser and a 561-nm laser. GFP and mCherry fluorescence was collected using a 525/50-nm emission filter and a 595/50-nm emission filter, respectively. HEK293T cells were bathed in a chamber containing Tyrode’s solution. To measure the sensor kinetics, a glass pipette was positioned close to the sensor-expressing cells and the fluorescence signals were measured using confocal high-speed line scanning mode (scanning speed 1024 Hz). For on kinetics, 100 μM 5-HT was puffed from the pipette. For off kinetics, 100 μM HTR6 antagonist SB 271046 was puffed onto cells bathed in 1 μM 5-HT. The basal fluorescent intensity of GRAB-HTR6-PM before 5-HT applications was used as F_0_ for calculating the ‘on’ response, while the fluorescence intensity under 1 μM 5-HT was set as F_0_ for calculating the ‘off’ response.

#### Measurements of the GRAB-HTR6-cilia sensor

##### GRAB-HTR6-cilia titration curve

Serum deprived, JF552 labeled RPE-1 cells stably expressing GRAB-HTR6-cilia were imaged in a HEPES-buffered imaging media (140 mM NaCl, 20 mM HEPES, 2.5 mM KCl, 1.8 mM CaCl_2_, 1.0 mM MgCl_2_, pH = 7.4, mOsm = 300; Live Cell Imaging Solution, Thermo Fisher Scientific A14291DJ) using a 20x air Plan-Apochromat objective (Zeiss, NA = 0.8) with FAST Airyscan on a Zeiss 880 confocal microscope at 37°C. GFP and JF552 were excited with 488 nm and 561 nm lasers, respectively. Keeping the same field of view, z stacks were acquired at different concentrations of 5-HT diluted in the same imaging buffer.

##### GRAB-HTR6-cilia with optogenetic stimulation

Hippocampal neurons expressing GRAB-HTR6-cilia and serotonergic neurons expressing ChrimsonR-tdTomato, farnesylated SNAP-Tag:JF552, or tdTomato were imaged with a 40x multi-immersion LD LCI Plan-Apochromat objective (Zeiss, NA = 1.2; silicone oil was used as the immersion media) on a Zeiss LSM 880 microscope in artificial cerebral spinal fluid (NaCl 124 mM, KCl 2.5 mM, NaH_2_PO_4_ 1.2 mM, NaHCO_3_ 24 mM, HEPES 5 mM, glucose 12.5 mM, MgSO_4_ 2mM, CaCl_2_ 2mM, Ting et al., 2018) at 37°C. Two-channel FAST Airyscan images were first imaged to characterize the axociliary synapses using 488 nm or 561 nm excitations for GFP and red fluorophores, respectively. GRAB-HTR6-cilia was then imaged at 1 Hz using the 488 nm laser with z-stacks. After 30 s, the 594 nm laser line was used to photostimulate at the same 1 Hz frequency (2 mW, 25 μm x 25 μm square, repetition = 1, pixel dwell time = 0.55 μs). A total of 120 z-stacks were acquired per cilium (120 s). Airyscan stacks were processed using Zen Black (auto-strength, 3D; Zeiss).

##### GRAB-HTR6-cilia with chemogenetic stimulation

Hippocampal neurons expressing GRAB-HTR6-cilia and serotonergic neurons expressing hM3Dq or farnesylated SNAP-Tag:JF552 were imaged with a 40x multi-immersion LD LCI Plan-Apochromat objective (Zeiss, NA = 1.2; silicone oil was used as the immersion media) on a Zeiss LSM 880 microscope in artificial cerebral spinal fluid (NaCl 124 mM, KCl 2.5 mM, NaH_2_PO_4_ 1.2 mM, NaHCO_3_ 24 mM, HEPES 5 mM, glucose 12.5 mM, MgSO_4_ 2mM, CaCl_2_ 2mM, Ting et al., 2018) at 37°C. Two-channel FAST Airyscan images were first imaged to characterize the axociliary synapses using 488 nm, 561 nm, or 594 nm excitations for GFP, JF552, and mCherry, respectively. GRAB-HTR6-cilia were then imaged at every 5 s using the 488 nm laser with z-stacks. After 1 min, 10 nM DCZ was directly added to the imaging media. A total of 60 z-stacks were acquired per cilium (3 min).

#### HTR6-RhoA FRET measurements

Images were collected using silicone-oil immersion media with a 40x multi-immersion LD LCI Plan-Apochromat objective (Zeiss, NA = 1.2; silicone oil immersion media) on a Zeiss LSM 880 microscope. Single apical cilia were imaged at zoom 10 with 4 Airy units (146 μm pinhole), 212 x 212 frame size, 0.1 μm pixel size, 0.93 μs pixel dwell time, 16-bit bidirectional scanning, and 4 optical sections (1.8 μm) to cover the entire length of the cilium. The resulting temporal resolution was 0.25 s. The donor fluorophore, sGFP2 was excited by the 488 nm laser. Donor emission from mScarlet-I was collected with the spectral detector set to 490-550 nm. Acceptor-sensitized emission was collected with the spectral detector set to 570-650 nm.

For uncaging experiments, the following uncaging parameters were used on a 5 μm radius circle adjacent to the ciliary tip: repeat each stack x10 using the same pixel dwell time as during scanning. Two experiment blocks (400 time frames each) were used to acquire data with and without uncaging. Uncaging started at frame 20 in the first block, with no uncaging during the second block.

#### Ciliary-RhoA FLIM measurements

3D z-stacks covering entire cilia were collected at 5 min intervals using an 40x oil immersion Plan-Apochromat objective (Leica, NA = 1.3) on a Leica SP8 Falcon microscope at 2x the Nyquist limit and 1 Airy disc. Donor sGFP fluorescence was excited by a pulsed (40 MHz) white light laser tuned at 488 nm, and emitted photons between 490 nm and 550 nm were collected with line repetition = 8.

#### ATAC-see staining in brain sections

Transposase-mixture solution (hyperactive Tn5 transposase with ATTO-590 conjugated oligos) were prepared as described previously (Chen et al., 2016). 50 μm thick, PFA-fixed CA1 coronal sections from 4-month-old C57BL/6 wild-type, *Htr6* KO, non-doxycycline-treated control, and doxycycline-treated HTR6-TRIP mice were obtained using the same method as described above. A 3 mm punch of the CA1 area was created using a biopsy punch on the 50 μm thick sections (Electron Microscopy Sciences). These punches were permeabilized in 0.3% Triton/PBS overnight at RT followed by 0.1% Triton, 0.1% Tween-20, and 0.01% digitonin in 10 mM Tris-HCL, 1 mM NaCl, 1.5 mM MgCl_2_ buffer (ATAC-RSB, or ATAC-Resuspension buffer) for 2 hrs. The samples were then rinsed in 0.1% Tween-20 in ATAC-RSB for 3 times and incubated in the transposase-mixture solution (100 nM Tn5 transposase, 10 mM Tris-HCl pH 7.5, 5 mM MgCl_2_, 33% PBS, 0.01% digitonin, 0.1% Tween-20, 8% PEG8000) for 2 h at 37°C on a shaker. After incubation, the samples were washed x 3 for 15 min at 55 °C with 1 × PBS containing 0.01% SDS and 50 mM EDTA, followed by regular PBS at room temperature for 15 min. The samples were subsequently stained with Hoechst 33342 (10 ug/ml) for 2 h, rinsed in PBS 15, and mounted in Slow Fade Glass (Thermo Fisher Scientific). Non-doxycycline-treated control and doxycycline-treated HTR6-TRIP samples were also stained with ADCY3 and CF633.

### QUANTIFICATION AND STATISTICAL ANALYSIS

Quantification and statistical analyses of GRAB-HTR6 and ciliary RhoA FRET/FLIM were described above in their respective sections. Details of experiments, including sample size and statistics can be found in the text, figures, or figure legends. Two mice per condition were used in Figures 6, 7, S3C, S3D, S6, and S7. Except for Figures 3E, S3C, S3D, and S6, all statistical tests were done using estimation statistics (Ho et al., 2019). Estimation statistics focuses on the effect size and its precision. In all estimation statistics plots, the effect size, or the mean difference, is presented as a bootstrap sampling distribution with 5000 bootstrap samples. In most cases, conventional p-values using nonparametric tests were also provided. No method was used to predetermine sample size, which represents data collected spanning different sessions. Blinding was not performed. Formal randomization techniques were not used.

#### Quantification of cilia trajectory in the brain

3D stacks of ADCY3-stained cilia images were projected along the z axis using maximum intensity projections and analyzed with the OrientationJ plug-in in ImageJ/Fiji using a gaussian gradient (Püspöki et al., 2016; Rezakhaniha et al., 2012). The algorithm computes the structure tensor for each pixel in the image using a sliding gaussian analysis window. A local window size of 2 and 20 pixels are used for visualization and weighted histogram calculation, respectively. Gaussian fitting of the weighted histogram was performed and plotted in Graphpad Prism (v9.2).

#### Quantification of cilia and serotonergic axon, synaptophysin vesicle apposition and nuclear adducin puncta

##### Linear Unmixing

3D stacks of Hoechst-stained nuclei, SERT-stained serotonergic axons (CF488), synaptophysin-stained presynaptic terminals (Alexa Fluor Plus 555), and ADCY3-stained cilia (CF633) images were acquired using FAST Airyscan. The red and far-red channel Airyscan data (41 nm x 41 nm x 189 nm voxel size, 3D Airyscan processing with Auto Filter) were unmixed via a custom MATLAB script.

##### Nucleus, cilia, axon and synaptophysin segmentation

The multichannel volumes acquired were resampled to generated isotropic voxel sizes of 189 nm. The nucleus, cilia, and axon signals were segmented using Otsu’s methods to threshold, followed by dilation and erosion operations to join fragmented structures, and finally filtered to remove small discrete objects with no biological relevance. Nuclei that were not part of the pyramidal neuron cell layer were removed by dilating the dense collection of nuclei, and subsequently retaining just the largest connected object (corresponding to pyramidal neuron cell layer). Only nuclei and associated cilia with the pyramidal neuron cell layer were included for downstream analysis steps. The central axes of segmented cilia and axon masks were computed (skeletonized), which also served to separate and identify distinct cilia. The cilia near the imaged volume boundaries or those which were associated with non-pyramidal neuron cell layer nuclei were excluded from further analyses. The synaptophysin and ADD1 puncta positions were determined by detecting their local signal maxima using a 3D Laplacian-of-Gaussian filter described previously (Aguet et al., 2016). Nucleus centers were determined by eroding the segmented nuclei masks, followed by calculating the distance transformation and thresholded to separate touching nuclei. The centroids of these discrete eroded nuclei were used to define the centers of search for ADD1 puncta with varying radii between 1 - 8 μm.

##### Axon-cilia and SERT+ synaptophysin-cilia distance

3D distance transformations of the nuclei, skeletonized axon, and synaptophysin masks were calculated and multiplied by the skeletonized cilia masks. The resulting mask encoded the distance information for the respective transformation mask in the medial axes of the cilia. As serotonergic varicosities are between 1 and 3 μm (Alvarez et al., 1998), we used 2 μm between skeletonized cilia and skeletonized axons as the cutoff for axon-contacting and non-axon-contacting cilia. Subsequently, synaptophysin puncta within 1 μm of skeletonized serotonergic axon central axes are considered associated with serotonergic axons (SERT-positive). For the visualization of the distance-encoded cilia, the cilia central axes were dilated using a sphere morphological structural kernel with a radius of 1 pixel. The resulting volumes were then projected to only visualize the distance information using a custom lookup table (Figure 2C). Furthermore, the total cilia length and the corresponding distance to the closest axon was plotted as a 2D density scatter plot using MATLAB (Chung, 2021) with a marker size set to 10 and a “jet” colormap (Figure S3C). Violin plots of cilium length for contacting and non-contacting cilia (Figure S3D) were generated using PlotsOfData (Postma and Goedhart, 2019). Statistical comparison of the two groups were done in GraphPad Prism. Permutation tests to compare these two distributions were performed using Mlxtend in Python (Raschka, 2018).

#### Quantification of ciliary 5-HTR6

Confocal datasets collected on Leica Stellaris (0.053 μm x 0.053 μm x 0.311 μm) were first downsampled to achieve isotropic voxels (0.311 μm). ADCY3 signals were first used to create ciliary masks by Otsu threshold method followed by dilation by 1 pixel. These masks were applied to the 5-HTR6_CF633 channel (2.3 ns) to calculate the integrated intensity of ciliary 5-HTR6_CF633. Total integrated intensity of 5_HTR6_CF633 was calculated by first thresholding using photon count 20 as the cutoff. The integrated ciliary 5-HTR6_CF633 intensity was then divided by the total 5-HTR6-CF633 integrated intensity to obtain the proportion of 5-HTR6 in the cilia.

#### Quantification of GRAB-HTR6 sensor measurements

##### Plasma membrane-located GRAB-HTR6-PM sensor

Images collected by the Opera Phenix high-content screening system from cultured HEK293T cells were processed using ImageJ (1.53c) software (NIH) and analyzed using custom-written MATLAB (R2020b) codes. The fluorescence response (ΔF/F_0_) was calculated using the formula (F–F_0_)/F_0_, in which F_0_ is the baseline fluorescence signal after subtracting the background. The dose-response curve was plotted using OriginPro (2020b). On and off kinetics were calculated based on the exponential-function fitting in OriginPro (2020b). For specificity analysis, the fluorescence responses (ΔF/F_0_) to different chemicals were corrected by subtracting the response to vehicle.

##### Titration curve of GRAB-HTR6-cilia sensor

The two channel images were first aligned using Zen Blue (Zeiss). Cilia were segmented using CiliaQ based on the JF552 channel (Hansen et al., 2021). Per cilium GFP mean fluorescent intensities were then calculated for different concentrations. The fluorescence response (ΔF/F_0_) was calculated using the formula (F–F_0_)/F_0_, in which F_0_ is the baseline fluorescence signal after subtracting the background. The dose-response curve was plotted using Graphpad Prism 9.2.

##### GRAB-HTR6-cilia with optogenetic stimulation

The 4D stacks (xyzt) were projected onto single planes (xyt) by maximum intensity projection in Fiji/ImageJ. Cilia were segmented using CiliaQ (Hansen et al., 2021). The first 10 time points, which often showed significant quenching, were discarded. A 1-μm circle at the contact site (ChrimsonR and non-ChrimsonR contacting cilia controls) or at the site at which the cilium is closest to a serotonergic axon (ChrimsonR non-contacting cilia) was used as the region of interest (ROI) to calculate mean intensity values over time after background substraction, which were then filtered with a 0.2 Hz low pass filter. The average intensity of the 10 time points prior to photostimulation was used as the baseline fluorescence intensity, or F_0_. The maximum, or peak ΔF/F_0_ during photostimulation was used for statistical comparisons in different conditions using estimation statistics (Ho et al., 2019). Low pass filtering, baseline fluorescence intensity calculations, and estimation statistics were done in a custom Python code. Traces of the time course were plotted using PlotTwist (Goedhart, 2020).

##### GRAB-HTR6-cilia with chemogenetic stimulation

The 4D stacks (xyzt) were projected onto single planes (xyt) by maximum intensity projection in Fiji/ImageJ, and downsampled temporally by a factor of 2 to obtain one stack per 10 s (30 total stacks). Airyscan stacks were processed using Zen Black (auto-strength, 3D; Zeiss). Cilia were segmented using CiliaQ (Hansen et al., 2021). A 1-μm circle at the contact site (hM3Dq or SNAP:JF522) or at the site at which the cilium is closest to a serotonergic axon (non-contacting hM3Dq) was used as the region of interest (ROI) to calculate mean intensity values over time after background substraction (ImageJ/Fiji). The average intensity of the first 5 time points prior to ligand application was used as the baseline fluorescence intensity, or F_0_. The average ΔF/F_0_ during ligand stimulation was used for statistical comparisons in different conditions using estimation statistics (Ho et al., 2019). Fluorescence intensity calculation, average ΔF/F_0_ and estimation statistics were done using a custom Python code. Traces of the time course were plotted using PlotTwist (Goedhart, 2020).

#### HTR6-RhoA FRET analysis

The 4D stacks (xyzt) were first projected onto single planes (xyt) by maximum intensity projection in Fiji/ImageJ. Datasets with bidirectional scanning artefacts (i.e., misalignment between two scanning directions in alternating lines), significant movement, and/or focus drifts were excluded. The donor channel was used to create a mask to segment the cilia (“cilia mask”) by using the “subtract background with smoothing paraboloid” command followed by Otsu thresholding in Fiji/ImageJ. The donor and sensitized emission intensity values were individually subtracted by using the mean intensity from an acellular area outside the cilium. These background-subtracted images were then masked using the cilia mask to isolate/segment cilia-specific signals. The FRET ratio was calculated by dividing the segmented sensitized emission by segmented donor emission. For direct ligand application experiments, FRET recordings were digitized at 4 Hz and filtered at 1 Hz with a low pass Fourier transform digital filter implemented in Origin (OriginLab Corporation). For uncaging experiments, the FRET data was processed with temporal averaging by a factor of 2. Change of the mean FRET ratio of the entire cilium was plotted using the Z plot function in Fiji/ImageJ and imported into Excel spreadsheets. The changes were visualized using PlotTwist (Goedhart, 2020). ΔF/F_0_ was calculated by using the mean FRET ratio at the beginning of the experiments as the baseline (frame 1 to 25 for direct ligand application, and 1 to 30 for uncaging experiments). Statistical testing of RhoA spikes were done in Prism 8 (GraphPad).

#### Ciliary-RhoA FLIM analysis

To calculate the fluorescence lifetime of unquenched and quenched donor, large populations of cells expressing the RhoA sensor were imaged, and fluorescence lifetimes were calculated by the n-exponential reconvolution fitting algorithm (2 exponential components) with pixel binning by a factor of 2 (Leica LAS X FLIM/FCS software v3.5). The fluorescence lifetime of quenched and unquenched donor was determined to be 1.3 and 2.7 ns, respectively. These numbers were then used for all subsequent fittings. To calculate representative RhoA sensor sGFP fluorescence lifetime per cilium, “FLIM Image Fit” was performed in the Leica LAS X FLIM/FCS software suite. The resulting datasets were rendered in 3D for visualization and exported as two-channel stacks, encoding photon counts and fluorescence lifetime, respectively. Subsequent imaging analyses were done in Python. For each stack, the channel encoding counts were used to segment cilia (Otsu and Yen thresholding were used for HEK cells and neurons, respectively). The segmented cilia were then used as masks to extract ciliary voxels from the FLIM channel. Voxels with less than 50 counts were discarded. An alpha distribution was fitted to the histogram of the FLIM channel, as it provided the best fitting among all 80 different statistical distributions tested. The peak of the alpha distribution was used as the mode, or the representative FLIM of a given cilium. Statistical comparisons of cilia from different cell types and states, and after stimulation were performed using estimation statistics (Ho et al., 2019).

#### Quantification of histone acetylation and ATAC-see

Airyscan stacks (0.035 μm x 0.035 μm x 0.15 μm) were first downsampled to get isotropic voxels (0.15 μm x 0.15 μm x 0.15 μm). The Hoechst channel was then used to segment nuclei. Histone marker stainings and ATAC were divided against Hoechst intensity on a per voxel basis to obtain histone-to-DNA or ATAC-to-DNA ratios. To account for the depth dependence on the antibody penetration, a flat-field correction was performed prior to calculating intensity ratios. The flat field was calculated by taking the mean projection through the y axis to generate x-z planes for each antibody-stained channel. Each channel was first normalized then averaged. To prevent sudden variations in intensity (since the flat field was calculated using the 3D volumes), a 2D Gaussian filter (sigma=2) was applied and the resulting flat field image was averaged through the x axis, then normalized by the resulting max value to generate the z-plane intensity correction vector. Each antibody treated channel was normalized by the calculated flat field correction vector. Hoechst channel background was calculated using Otsu’s method where the input signal values to calculate the threshold were restricted to 90^th^ percentile of the image data. The ratio was not calculated in voxels where the Hoechst signal was lower than Otsu’s threshold. The stack histograms were plotted in Python with kernel density estimates. The mode of each condition is the peak of the kernel density estimate.

## Supplementary Material

Video S1

Methods S1

3

## Figures and Tables

**Table:** KEY RESOURCES TABLE

REAGENT or RESOURCE	SOURCE	IDENTIFIER
Antibodies		
Rabbit polyclonal anti-GFP	Chromotek	Cat# PABG1; RRID:AB_2749857
Guinea pig polyclonal anti-SERT	Synaptic Systems	Cat# 340 004; RRID:AB_2620086
Mouse monoclonal anti-ADCY3	Encor Biotechnology	Cat# MCA-1A12; RRID:AB_2744501
Rabbit anti-PCP4	Millipore Sigma	Cat# HPA005792; RRID:AB_1855086
Chicken anti-rootletin	Millipore Sigma	Cat# ABN1686; RRID:AB_2893142
Rabbit anti-ADD1	Abcam	Cat# ab40760; RRID:AB_722627
Rabbit anti-synaptophysin	Cell Signaling	Cat# 36406; RRID:AB_2799098
Rabbit anti-synaptophysin	Thermo Fisher Scientific	Cat# MA5-14532; RRID:AB_10983675
Rabbit anti-Trio GEFD2	Laboratory of Dr. Susanne Schmidt	N/A
Rabbit anti-h4k5ac	Thermo Fisher Scientific	Cat# MA5-32009; RRID:AB_2809303
Mouse anti-panh4ac	Thermo Fisher Scientific	Cat# MA3-066; RRID:AB_2633028
Rabbit anti-H3K27ac	Abcam	Cat# ab177178; RRID:AB_2828007
Alexa Fluor Plus 488 goat anti-rabbit	Thermo Fisher Scientific	Cat# A32731; RRID:AB_2633280
CF488A donkey anti-guinea pig	Biotium	Cat# 20169; RRID:AB_10853115
Alexa Fluor Plus 555 goat anti-rabbit	Thermo Fisher Scientific	Cat# A32732; RRID:AB_2633281
CF555 goat anti-mouse IgG1	Biotium	Cat# 20247; RRID:AB_10854998
CF633 goat anti-mouse IgG_1_	Biotium	Cat# 20250; RRID:AB_10852830
CF633 donkey anti-rabbit	Biotium	Cat# 20215; RRID:AB_10853935
Bacterial and virus strains		
pAAV-Syn-FLEX-rc[ChrimsonR-tdTomato]	HHMI Viral Tools	N/A
pAAV[flex_on]-CAG-Farnesylated-SNAPtag	HHMI Viral Tools	N/A
pAAV-EF1a-DIO-hM3D(Gq)-mCherry	HHMI Viral Tools	N/A
pAAV-TRE-HTR6-SNAP-TRIP	HHMI Viral Tools	N/A
pAAV-SYN1-Tet3G	HHMI Viral Tools	N/A
pAAV phSyn1(S)-FLEX-tdTomato-T2A-SypEGFP-WPRE	Addgene	Addgene viral prep # 51509-AAV1
Biological samples		
Hippocampal and raphe neuron culture	In house	N/A
Chemicals, peptides, and recombinant proteins		
Tn5 transposase	In house	N/A
PA-O-Ser	This paper	N/A
SB 271046 hydrochloride	Tocris	Cat# 3368; CAS 209481-24-3
SB-258585 hydrochloride	Caymen Chemicals	Cat# 17416; CAS 1216468-02-8
WAY181187 oxalate	Tocris	Cat# 5589; CAS 1883548-85-3
H-89 hydrochloride	Caymen Chemicals	Cat# 10010556; CAS 130964-39-5
Hoechst 33342	Thermo Fisher Scientific	Cat# 62249; CAS 875756-97-1
Deschloroclozapine	Tocris	Cat# 7193; CAS 1977-07-7
5-hydroxytrptamine (serotonin, 5-HT)	Alfa Aesar	Cat# B21263-06; CAS 153-98-0
Acetylcholine chloride	Solarbio	Cat# G8320; CAS 60-31-1
Adenosine	Sigma-Aldrich	Cat# A4036; CAS 58-61-7
Adenosine 5’-triphosphate (ATP)	Sigma-Aldrich	Cat# A7699; CAS 34369-07-8
Dopamine hydrochloride	Sigma-Aldrich	Cat# H8502; CAS 62-31-7
γ-Aminobutyric acid (GABA)	Tocris	Cat# 0344; CAS 56-12-2
L-Glutamic acid	Sigma-Aldrich	Cat# V900408; CAS 56-86-0
Glycine	Sigma-Aldrich	Cat# G7403; CAS 56-40-6
Histamine dihydrochloride	Tocris	Cat# 3545; CAS 56-92-8
Melatonin	Sigma-Aldrich	Cat# M5250; CAS 73-31-4
Norepinephrine bitartrate	Tocris	Cat# 5169; CAS 51-40-1
Octopamine hydrochloride	Abcam	Cat# ab120770; CAS 770-05-8
Tyramine	Aladdin	Cat# T105543; CAS 51-67-2
L-Tryptophan	Sigma-Aldrich	Cat# 93659; CAS 73-22-3
Critical commercial assays		
Alexa Fluor^™^ 488 Tyramide SuperBoost^™^ Kit, goat anti-rabbit IgG	Thermo Fisher Scientific	Cat# B40922
Experimental models: Cell lines		
hTERT RPE-1	ATCC	CRL-4000
HEK293A	Laboratory of Dr. Asuka Inoue, https://doi.org/10.1038/ncomms10156	N/A
HEK293A GNAQ/11 KO	Laboratory of Dr. Asuka Inoue, https://doi.org/10.1038/ncomms10156	N/A
HEK293T	ATCC	1573
Experimental models: Organisms/strains		
Mouse: C56BL/6	Charles River	Cat# 027; RRID:IMSR_CRL:027
Mouse: C57BL/6J	Jackson Laboratory	Cat# 000664; IMSR_JAX:000664
Mouse: *Htr6* KO	This paper	N/A
Mouse H*tr6*-EGFP knock-in	Laboratory of Dr. Séverine Chaumont-Dubel, https://doi.org/10.1073/pnas.1600914113	N/A
Oligonucleotides		
[phos]CTGTCTCTTATACACATCT	Chen et al., 2016	N/A
/ATTO594/TCGTCGGCAGCGTCAGATGTGTATAAGAGACAG	Chen et al., 2016	N/A
/ATTO594/GTCTCGTGGGCTCGGAGATGTGTATAAGAGACAG	Chen et al., 2016	N/A
Recombinant DNA		
HTR6-Tango	Kroeze et al., 2015	Addgene 66414
Tet-On HTR6-RhoA sensor	This paper	N/A
Tet-On Arl13b-RhoA sensor	This paper	N/A
*Tph2*-Cre	This paper	N/A
pAAV-Syn-FLEX-rc[ChrimsonR-tdTomato	Klapoetke et al., 2014	Addgene 62723
pAAV[flex_on]-CAG-Farnesylated-SNAPtag	This paper	N/A
pAAV-FLEX-tdTomato	Unpublished; Laboratory of Ed Boyden	Addgene 28306
pAAV-EF1a-DIO-hM3D(Gq)-mCherry	Unpublished; Laboratory of Dr. Bryan Roth.	Addgene 50460
pAAV-TRE-HTR6-SNAP-TRIP	This paper	N/A
pAAV-SYN1-Tet3G	This paper	N/A
Tet-On GRAB-HTR6-cilia:3xGGGGS:Halo tag	This paper	N/A
GRAB-HTR6-PM	This paper	N/A
Hyperactive piggybac transposase	VectorBuilder	N/A
Software and algorithms		
ImageJ/Fiji	Schindelin et al., 2012	https://imagej.net/software/fiji/
Prism v9.2	Graphpad Software	https://www.graphpad.com
MATLAB 2020b, 2021a	The MathWorks	https://www.mathworks.com/
Python 3.8 (Anaconda)	Anaconda	https://www.anaconda.com/
DABEST	Ho et al., 2019	https://acclab.github.io/DABEST-python-docs/index.html
OrientationJ	Püspöki et al., 2016; Rezakhaniha et al., 2012	https://github.com/Biomedical-Imaging-Group/OrientationJ
VAST Lite	Berger et al., 2018	https://lichtman.rc.fas.harvard.edu/vast/
3ds Max 2021	Autodesk	https://www.autodesk.com/products/3ds-max/overview
Mlextend	Raschka, 2018	http://rasbt.github.io/mlxtend/

## Highlights

- Neuronal axons can form synapses with primary cilia
- Opto- and chemogenetic stimulation of serotonergic axons releases serotonin onto cilia
- Ciliary 5-HTR6 stimulation activates a non-canonical G_αq/11_-RhoA pathway
- Ciliary RhoA modulates nuclear actin, histone acetylation, and chromatin accessibility

## References

[R1] Aguet F, Upadhyayula S, Gaudin R, Chou YY, Cocucci E, He K, Chen B-C, Mosaliganti K, Pasham M, Skillern W, (2016). Membrane dynamics of dividing cells imaged by lattice light-sheet microscopy. Mol. Biol. Cell 27, 3418–3435. 10.1091/mbc.E16-03-0164. 27535432 PMC5221578

[R2] Alvarez FJ, Pearson JC, Harrington D, Dewey D, Torbeck L, and Fyffe REW (1998). Distribution of 5-hydroxytryptamine-immunoreactive boutons on α-motoneurons in the lumbar spinal cord of adult cats. J. Comp. Neurol 393, 69–83. 10.1002/(sici)1096-9861(19980330)393:1&lt;69::aidcne7&gt;3.0.co;2-o. 9520102

[R3] Anvarian Z, Mykytyn K, Mukhopadhyay S, Pedersen LB, and Christensen ST (2019). Cellular signalling by primary cilia in development, organ function and disease. Nat. Rev. Nephrol 15, 199–219. 10.1038/s41581-019-0116-9. 30733609 PMC6426138

[R4] Arellano JI, Guadiana SM, Breunig JJ, Rakic P, and Sarkisian MR (2012). Development and distribution of neuronal cilia in mouse neocortex. J. Comp. Neurol 520, 848–873. 10.1002/one.22793. 22020803 PMC3325766

[R5] Armbruster BN, Li X, Pausch MH, Herlitze S, and Roth BL (2007). Evolving the lock to fit the key to create a family of G protein-coupled receptors potently activated by an inert ligand. Proc. Natl. Acad. Sci. USA 104, 5163–5168. 10.1073/pnas.0700293104. 17360345 PMC1829280

[R6] Baldi P, Alhassen W, Chen S, Nguyen H, Khoudari M, and Alachkar A (2021). Large-scale analysis reveals spatiotemporal circadian patterns of cilia transcriptomes in the primate brain. J. Neurosci. Res 99, 2610–2624. 10.1002/jnr.24919. 34310750 PMC11391745

[R7] Barbeito P, Tachibana Y, Martin-Morales R, Moreno P, Mykytyn K, Kobayashi T, and Garcia-Gonzalo FR (2021). HTR6 and SSTR3 ciliary targeting relies on both IC3 loops and C-terminal tails. Life Sci. Alliance 4. e202000746. 10.26508/lsa.202000746. 33372037 PMC7772773

[R8] Belmer A, Klenowski PM, Patkar OL, and Bartlett SE (2017). Mapping the connectivity of serotonin transporter immunoreactive axons to excitatory and inhibitory neurochemical synapses in the mouse limbic brain. Brain Struct. Funct 222, 1297–1314. 10.1007/s00429-016-1278-x. 27485750 PMC5368196

[R9] Bennett V, and Gilligan DM (1993). The spectrin-based membrane skeleton and micron-scale organization of the plasma membrane. Annu. Rev. Cell Biol 9, 27–66. 10.1146/annurev.cb.09.110193.000331. 8280463

[R10] Benzekhroufa K, Liu B-H, Teschemacher AG, and Kasparov S (2009). Targeting central serotonergic neurons with lentiviral vectors based on a transcriptional amplification strategy. Gene Ther 16, 681–688. 10.1038/gt.2009.7. 19212426

[R11] Berbari NF, Johnson AD, Lewis JS, Askwith CC, and Mykytyn K (2008). Identification of ciliary localization sequences within the third intracellular loop of G protein-coupled receptors. MBoC 19, 1540–1547. 10.1091/mbc.e07-09-0942. 18256283 PMC2291422

[R12] Berger DR, Seung HS, and Lichtman JW (2018). VAST (Volume Annotation and Segmentation Tool): efficient manual and semi-automatic labeling of large 3D image stacks. Front. Neural Circuits 12, 88. 10.3389/fncir.2018.00088. 30386216 PMC6198149

[R13] Bindels DS, Haarbosch L, Weeren L. van, Postma M, Wiese KE, Mastop M, Aumonier S, Gotthard G, Royant A, Hink MA, (2017). mScarlet: a bright monomeric red fluorescent protein for cellular imaging. Nat. Methods 14, 53–56. 10.1038/nmeth.4074. 27869816

[R14] Bishop GA, Berbari NF, Lewis J, and Mykytyn K (2007). Type III adenylyl cyclase localizes to primary cilia throughout the adult mouse brain. J. Comp. Neurol 505, 562–571. 10.1002/cne.21510. 17924533

[R15] Boess FG, Monsma FJ, Carolo C, Meyer V, Rudler A, Zwingelstein C, and Sleight AJ (1997). Functional and radioligand binding characterization of rat 5-HT 6 receptors stably expressed in HEK293 Cells. Neuropharmacology 36, 713–720. 10.1016/S0028-3908(97)00019-1. 9225298

[R16] Bouquier N, Fromont S, Zeeh J-C, Auziol C, Larrousse P, Robert B, Zeghouf M, Cherfils J, Debant A, and Schmidt S (2009). Aptamer-derived peptides as potent inhibitors of the oncogenic RhoGEF Tgat. Chem. Biol 16, 391–400. 10.1016/j.chembiol.2009.02.006. 19389625

[R17] Brodsky M, Lesiak AJ, Croicu A, Cohenca N, Sullivan JM, and Neumaier JF (2017). 5-HT6 receptor blockade regulates primary cilia morphology in striatal neurons. Brain Res 1660, 10–19. 10.1016/j.brainres.2017.01.010. 28087224 PMC5392252

[R18] Chen C-L, Lin Y-P, Lai Y-C, and Chen H-C (2011). α-adducin translocates to the nucleus upon loss of cell-cell adhesions. Traffic 12, 1327–1340. 10.1111/j.1600-0854.2011.01245.x. 21736685

[R19] Chen X, Shen Y, Draper W, Buenrostro JD, Litzenburger U, Cho SW, Satpathy AT, Carter AC, Ghosh RP, East-Seletsky A, (2016). ATAC-see reveals the accessible genome by transposase-mediated imaging and sequencing. Nat. Methods 13, 1013–1020. 10.1038/nmeth.4031. 27749837 PMC5509561

[R20] Chung M (2021). Density scatter plot–file exchange–MATLAB Central. https://www.mathworks.com/matlabcentral/fileexchange/56569-density-scatter-plot.

[R21] Cole DC, Stock JR, Lennox WJ, Bernotas RC, Ellingboe JW, Boikess S, Coupet J, Smith DL, Leung L, Zhang G-M, (2007). Discovery of N1-(6-Chloroimidazo[2, 1-b] [1, 3]thiazole-5-sulfonyl)tryptamine as a potent, selective, and orally active 5-HT6 receptor agonist. J. Med. Chem 50, 5535–5538. 10.1021/jm070521y. 17948978

[R22] Corces MR, Trevino AE, Hamilton EG, Greenside PG, Sinnott-Armstrong NA, Vesuna S, Satpathy AT, Rubin AJ, Montine KS, Wu B, (2017). An improved ATAC-seq protocol reduces background and enables interrogation of frozen tissues. Nat. Methods 14, 959–962. 10.1038/nmeth.4396. 28846090 PMC5623106

[R23] Deo C, Sheu S-H, Seo J, Clapham DE, and Lavis LD (2019). Isomeric tuning yields bright and targetable red Ca^2+^ indicators. J. Am. Chem. Soc 141, 13734–13738. 10.1021/jacs.9b06092. 31430138

[R24] Nadim W, Chaumont-Dubel S, Madouri F, Cobret L, Tauzia M-L, Zajdel P, Bénédetti H, Marin P, and Morisset-Lopez S (2016). Physical interaction between neurofibromin and serotonin 5-HT6 receptor promotes receptor constitutive activity. Proc. Natl. Acad. Sci. USA 113, 12310–12315. 10.1073/pnas.1600914113. 27791021 PMC5087017

[R25] Domire JS, Green JA, Lee KG, Johnson AD, Askwith CC, and Mykytyn K (2011). Dopamine receptor 1 localizes to neuronal cilia in a dynamic process that requires the Bardet-Biedl syndrome proteins. Cell. Mol. Life Sci 68, 2951–2960. 10.1007/s00018-010-0603-4. 21152952 PMC3368249

[R26] Douanne T, Stinchcombe JC, and Griffiths GM (2021). Teasing out function from morphology: similarities between primary cilia and immune synapses. J. Cell Biol 220. e202102089. 10.1083/jcb.202102089. 33956049 PMC8105739

[R27] Einstein EB, Patterson CA, Hon BJ, Regan KA, Reddi J, Melnikoff DE, Mateer MJ, Schulz S, Johnson BN, and Tallent MK (2010). Somatostatin signaling in neuronal cilia is critical for object recognition memory. J. Neurosci 30, 4306–4314. 10.1523/JNEUROSCI.5295-09.2010. 20335466 PMC3842454

[R28] Feng J, Zhang C, Lischinsky JE, Jing M, Zhou J, Wang H, Zhang Y, Dong A, Wu Z, Wu H, (2019). A genetically encoded fluorescent sensor for rapid and specific in vivo detection of norepinephrine. Neuron 102, 745–761.e8. 10.1016/j.neuron.2019.02.037. 30922875 PMC6533151

[R29] Feng X, Degese MS, Iglesias-Bartolome R, Vaque JP, Molinolo AA, Rodrigues M, Zaidi MR, Ksander BR, Merlino G, Sodhi A, (2014). Hippo-independent activation of YAP by the GNAQ uveal melanoma oncogene through a Trio-Regulated Rho GTPase signaling circuitry. Cancer Cell 25, 831–845. 10.1016/j.ccr.2014.04.016. 24882515 PMC4074519

[R30] Flatau G, Lemichez E, Gauthier M, Chardin P, Paris S, Fiorentini C, and Boquet P (1997). Toxin-induced activation of the G protein p21 Rho by deamidation of glutamine. Nature 387, 729–733. 10.1038/42743. 9192901

[R31] Fukata Y, Oshiro N, Kinoshita N, Kawano Y, Matsuoka Y, Bennett V, Matsuura Y, and Kaibuchi K (1999). Phosphorylation of adducin by Rho-kinase plays a crucial role in cell motility. J. Cell Biol 145, 347–361. 10.1083/jcb.145.2.347. 10209029 PMC2133101

[R32] Gao R, Asano SM, Upadhyayula S, Pisarev I, Milkie DE, Liu T-L, Singh V, Graves A, Huynh GH, Zhao Y, (2019). Cortical column and whole-brain imaging with molecular contrast and nanoscale resolution. Science 363, eaau8302. 10.1126/science.aau8302. 30655415 PMC6481610

[R33] Goedhart J (2020). PlotTwist: a web app for plotting and annotating continuous data. PLoS Biol 18, e3000581. 10.1371/journal.pbio.3000581. 31929523 PMC6980690

[R34] Goetz SC, and Anderson KV (2010). The primary cilium: a signalling centre during vertebrate development. Nat. Rev. Genet 11, 331–344. 10.1038/nrg2774. 20395968 PMC3121168

[R35] Grimm JB, English BP, Chen J, Slaughter JP, Zhang Z, Revyakin A, Patel R, Macklin JJ, Normanno D, Singer RH, (2015). A general method to improve fluorophores for live-cell and single-molecule microscopy. Nat. Methods 12, 244–250. 10.1038/nmeth.3256. 25599551 PMC4344395

[R36] Guemez-Gamboa A, Coufal NG, and Gleeson JG (2014). Primary cilia in the developing and mature brain. Neuron 82, 511–521. 10.1016/j.neuron.2014.04.024. 24811376 PMC4104280

[R37] Hagen V, Dekowski B, Kotzur N, Lechler R, Wiesner B, Briand B, and Beyermann M (2008). {7-[Bis(carboxymethyl)amino]coumarin-4-yl}methoxycarbonyl derivatives for photorelease of carboxylic acids, Alcohols/Phenols, Thioalcohols/Thiophenols, and Amines. Chem. Eur. J 14, 1621–1627. 10.1002/chem.200701142. 18046693

[R38] Han B, Zhou R, Xia C, and Zhuang X (2017). Structural organization of the actin-spectrin-based membrane skeleton in dendrites and soma of neurons. Proc. Natl. Acad. Sci. USA 114, E6678–E6685. 10.1073/pnas.1705043114. 28739933 PMC5559029

[R39] Hansen JN, Rassmann S, Stüven B, Jurisch-Yaksi N, and Wachten D (2021). CiliaQ: a simple, open-source software for automated quantification of ciliary morphology and fluorescence in 2D, 3D, and 4-D images. Eur. Phys. J. E Soft Matter 44, 18. 10.1140/epje/s10189-021-00031-y. 33683488 PMC7940315

[R40] Harkes R, Kukk O, Mukherjee S, Klarenbeek J, Broek B. van den, and Jalink K (2021). Dynamic FRET-FLIM based screening of signal transduction pathways. Sci. Rep 11, 20711. 10.1038/s41598-021-00098-9. 34671065 PMC8528867

[R41] Hilgendorf KI, Johnson CT, and Jackson PK (2016). The primary cilium as a cellular receiver: organizing ciliary GPCR signaling. Curr. Opin. Cell Biol 39, 84–92. 10.1016/j.ceb.2016.02.008. 26926036 PMC4828300

[R42] Hilgendorf KI, Johnson CT, Mezger A, Rice SL, Norris AM, Demeter J, Greenleaf WJ, Reiter JF, Kopinke D, and Jackson PK (2019). Omega-3 fatty acids activate ciliary FFAR4 to control adipogenesis. Cell 179, 1289–1305.e21. 10.1016/j.cell.2019.11.005. 31761534 PMC7332222

[R43] Hirst WD, Minton JAL, Bromidge SM, Moss SF, Latter AJ, Riley G, Routledge C, Middlemiss DN, and Price GW (2000). Characterization of [125I]-SB-258585 binding to human recombinant and native 5-HT6 receptors in rat, pig and human brain tissue. Br. J. Pharmacol 130, 1597–1605. 10.1038/sj.bjp.0703458. 10928963 PMC1572217

[R44] Ho J, Tumkaya T, Aryal S, Choi H, and Claridge-Chang A (2019). Moving beyond P values: data analysis with estimation graphics. Nat. Methods 16, 565–566. 10.1038/s41592-019-0470-3. 31217592

[R45] Hor CN, Yeung J, Jan M, Emmenegger Y, Hubbard J, Xenarios I, Naef F, and Franken P (2019). Sleep–wake-driven and circadian contributions to daily rhythms in gene expression and chromatin accessibility in the murine cortex. Proc. Natl. Acad. Sci. USA 116, 25773–25783. 10.1073/pnas.1910590116. 31776259 PMC6925978

[R46] Ioannou MS, Jackson J, Sheu S-H, Chang C-L, Weigel AV, Liu H, Pasolli HA, Xu CS, Pang S, Matthies D, (2019). Neuron-astrocyte metabolic coupling protects against activity-induced fatty acid toxicity. Cell 177, 1522–1535.e14. 10.1016/j.cell.2019.04.001. 31130380

[R47] Jiang JY, Falcone JL, Curci S, and Hofer AM (2019). Direct visualization of cAMP signaling in primary cilia reveals up-regulation of ciliary GPCR activity following Hedgehog activation. Proc. Natl. Acad. Sci. USA 116, 12066–12071. 10.1073/pnas.1819730116. 31142652 PMC6575585

[R48] Jones PB, Rozkalne A, Meyer-Luehmann M, Spires-Jones TL, Makarova A, Kumar ATN, Berezovska O, Bacskai BB, and Hyman BT (2008). Two postprocessing techniques for the elimination of background autofluorescence for fluorescence lifetime imaging microscopy. J. Biomed. Opt 13, 014008. 10.1117/1.2837169. 18315366

[R49] Kasthuri N, Hayworth KJ, Berger DR, Schalek RL, Conchello JAA, Knowles-Barley S, Lee D, Vázquez-Reina A, Kaynig V, Jones TR, (2015). Saturated reconstruction of a volume of neocortex. Cell 162, 648–661. 10.1016/j.cell.2015.06.054. 26232230

[R50] Kirschen GW, Liu H, Lang T, Liang X, Ge S, and Xiong Q (2017). The radial organization of neuronal primary cilia is acutely disrupted by seizure and ischemic brain injury. Front. Biol 12, 124–138. 10.1007/s11515-017-1447-1. PMC5412953 28473847

[R51] Klapoetke NC, Murata Y, Kim SS, Pulver SR, Birdsey-Benson A, Cho YK, Morimoto TK, Chuong AS, Carpenter EJ, Tian Z, (2014). Independent optical excitation of distinct neural populations. Nat. Methods 11, 338–346. 10.1038/nmeth.2836. 24509633 PMC3943671

[R52] Kohli P, Höhne M, Jüngst C, Bertsch S, Ebert LK, Schauss AC, Benzing T, Rinschen MM, and Schermer B (2017). The ciliary membrane-associated proteome reveals actin-binding proteins as key components of cilia. EMBO Rep 18, 1521–1535. 10.15252/embr.201643846. 28710093 PMC5579364

[R53] Korogod N, Petersen CC, and Knott GW (2015). Ultrastructural analysisof adult mouse neocortex comparing aldehyde perfusion with cryo fixation. eLife 4, e05793. 10.7554/eLife.05793. 26259873 PMC4530226

[R54] Kroeze WK, Sassano MF, Huang XP, Lansu K, McCorvy JD, Giguère PM, Sciaky N, and Roth BL (2015). Presto-Tango as an open-source resource for interrogation of the druggable human GPCRome. Nat. Struct. Mol. Biol 22, 362–369. 10.1038/nsmb.3014. 25895059 PMC4424118

[R55] Lau C, Ng L, Thompson C, Pathak S, Kuan L, Jones A, and Hawrylycz M (2008). Exploration and visualization of gene expression with neuroanatomy in the adult mouse brain. BMC Bioinformatics 9, 153. 10.1186/1471-2105-9-153. 18366675 PMC2375125

[R56] Lehmenkühler A, Syková E, Svoboda J, Zilles K, and Nicholson C (1993). Extracellular space parameters in the rat neocortex and subcortical white matter during postnatal development determined by diffusion analysis. Neuroscience 55, 339–351. 10.1016/0306-4522(93)90503-8. 8377929

[R57] Liu C-M, Hsu W-H, Lin W-Y, and Chen H-C (2017). Adducin family proteins possess different nuclear export potentials. J. Biomed. Sci 24, 30. 10.1186/s12929-017-0333-0. 28490361 PMC5424492

[R58] Lowe DG (2004). Distinctive image features from scale-invariant keypoints. International Journal of Computer Vision 60, 91–110. 10.1023/B:VISI.0000029664.99615.94.

[R59] Marco A, Meharena HS, Dileep V, Raju RM, Davila-Velderrain J, Zhang AL, Adaikkan C, Young JZ, Gao F, Kellis M, (2020). Mapping the epigenomic and transcriptomic interplay during memory formation and recall in the hippocampal engram ensemble. Nat. Neurosci 23, 1606–1617. 10.1038/s41593-020-00717-0. 33020654 PMC7686266

[R60] Masyuk AI, Huang BQ, Radtke BN, Gajdos GB, Splinter PL, Masyuk TV, Gradilone SA, and LaRusso NF (2013). Ciliary subcellular localization of TGR5 determines the cholangiocyte functional response to bile acid signaling. Am. J. Physiol. Gastrointest. Liver Physiol 304, G1013–G1024. 10.1152/ajpgi.00383.2012. 23578785 PMC3680685

[R61] Mirvis M, Siemers KA, Nelson WJ, and Stearns TP (2019). Primary cilium loss in mammalian cells occurs predominantly by whole-cilium shedding. PLoS Biol 17, e3000381. 10.1371/journal.pbio.3000381. 31314751 PMC6699714

[R62] Monsma FJ, Shen Y, Ward RP, Hamblin MW, and Sibley DR (1993). Cloning and expression of a novel serotonin receptor with high affinity for tricyclic psychotropic drugs. Mol. Pharmacol 43, 320–327. 7680751

[R63] Mullins N, Forstner AJ, O’Connell KS, Coombes B, Coleman JRI, Qiao Z, Als TD, Bigdeli TB, Børte S, Bryois J, (2021). Genome-wide association study of more than 40, 000 bipolar disorder cases provides new insights into the underlying biology. Nat. Genet 53, 817–829. 10.1038/s41588-021-00857-4. 34002096 PMC8192451

[R64] Muzerelle A, Scotto-Lomassese S, Bernard JF, Soiza-Reilly M, and Gaspar P (2016). Conditional anterograde tracing reveals distinct targeting of individual serotonin cell groups (B5–B9) to the forebrain and brainstem. Brain Struct. Funct 221, 535–561. 10.1007/s00429-014-0924-4. 25403254 PMC4750555

[R65] Nagai Y, Miyakawa N, Takuwa H, Hori Y, Oyama K, Ji B, Takahashi M, Huang X-P, Slocum ST, DiBerto JF, (2020). Deschloroclozapine, a potent and selective chemogenetic actuator enables rapid neuronal and behavioral modulations in mice and monkeys. Nat. Neurosci 23, 1157–1167. 10.1038/s41593-020-0661-3. 32632286

[R66] Nager AR, Goldstein JS, Herranz-Pérez V, Portran D, Ye F, Garcia-Verdugo JM, and Nachury MV (2017). An actin network dispatches ciliary GPCRs into extracellular vesicles to modulate signaling. Cell 168, 252–263.e14. 10.1016/j.cell.2016.11.036. 28017328 PMC5235987

[R67] Nishimura A, Kitano K, Takasaki J, Taniguchi M, Mizuno N, Tago K, Hakoshima T, and Itoh H (2010). Structural basis for the specific inhibition of heterotrimeric Gq protein by a small molecule. Proc. Natl. Acad. Sci. USA 107, 13666–13671. 10.1073/pnas.1003553107. 20639466 PMC2922266

[R68] Oikonomou G, Altermatt M, Zhang RW, Coughlin GM, Montz C, Gradinaru V, and Prober DA (2019). The serotonergic raphe promote sleep in zebrafish and mice. Neuron 103, 686–701.e8. 10.1016/j.neuron.2019.05.038. 31248729 PMC6706304

[R69] Oishi A, Makita N, Sato J, and Iiri T (2012). Regulation of RhoA signaling by the cAMP-dependent phosphorylation of RhoGDIα. J. Biol. Chem 287, 38705–38715. 10.1074/jbc.M112.401547. 23012358 PMC3493914

[R70] Park CS, Rehrauer H, and Mansuy IM (2013). Genome-wide analysis of H4K5 acetylation associated with fear memory in mice. BMC Genomics 14, 539. 10.1186/1471-2164-14-539. 23927422 PMC3751108

[R71] Park Y-G, Sohn C, Chen R, McCue M, Yun D, Drummond GT, Ku T, Evans NB, Oak H, Trieu W, (2018). Protection of tissue physicochemical properties using polyfunctional crosslinkers. Nat. Biotechnol 37, 73–83. 10.1038/nbt.4281. PMC6579717 30556815

[R72] Picelli S, Björklund AK, Reinius B, Sagasser S, Winberg G, and Sandberg R (2014). Tn5 transposase and tagmentation procedures for massively scaled sequencing projects. Genome Res 24, 2033–2040. 10.1101/gr.177881.114. 25079858 PMC4248319

[R73] Plessner M, and Grosse R (2019). Dynamizing nuclear actin filaments. Curr. Opin. Cell Biol 56, 1–6. 10.1016/j.ceb.2018.08.005. 30193156

[R74] Postma M, and Goedhart J (2019). PlotsOfData–a web app for visualizing data together with their summaries. PLoS Biol. 17, e3000202. 10.1371/journal.pbio.3000202. 30917112 PMC6453475

[R75] Püspöki Z, Storath M, Sage D, and Unser M (2016). Transforms and operators for directional BioImage analysis: a survey. Adv. Anat. Embryol. Cell Biol 219, 69–93. 10.1007/978-3-319-28549-8_3. 27207363

[R76] Qiao J, Huang F, and Lum H (2003). PKA inhibits RhoA activation: a protection mechanism against endothelial barrier dysfunction. Am. J. Physiol.-Lung C 284, L972–L980. 10.1152/ajplung.00429.2002. 12588708

[R77] Rahman MA, Kim H, Lee KH, Yun H-M, Hong J-H, Kim Y, Choo H, Park M, and Rhim H (2017). 5-Hydroxytryptamine 6 receptor (5-HT6R)-mediated morphological changes via RhoA-dependent pathways. Mol. Cells 40, 495–502. 10.14348/molcells.2017.0080. 28681593 PMC5547219

[R78] Rasch B, and Born J (2013). About Sleep’s Role in Memory. Physiol. Rev 93, 681–766. 10.1152/physrev.00032.2012. 23589831 PMC3768102

[R79] Raschka S (2018). MLxtend: providing machine learning and data science utilities and extensions to Python’s scientific computing stack. J. Open Source Softw 3, 638. 10.21105/joss.00638.

[R80] Rezakhaniha R, Agianniotis A, Schrauwen JTC, Griffa A, Sage D, Bouten CVC, Vosse F.N. van de, Unser M, and Stergiopulos N (2012). Experimental investigation of collagen waviness and orientation in the arterial adventitia using confocal laser scanning microscopy. Biomech. Model. Mechanobiol 11, 461–473. 10.1007/s10237-011-0325-z. 21744269

[R81] Schechter LE, Lin Q, Smith DL, Zhang G, Shan Q, Platt B, Brandt MR, Dawson LA, Cole D, Bernotas R, (2008). Neuropharmacological profile of novel and selective 5-HT6 receptor agonists: WAY-181187 and WAY-208466. Neuropsychopharmacology 33, 1323–1335. 10.1038/sj.npp.1301503. 17625499

[R82] Schindelin J, Arganda-Carreras I, Frise E, Kaynig V, Longair M, Pietzsch T, Preibisch S, Rueden C, Saalfeld S, Schmid B, (2012). Fiji: an open-source platform for biological-image analysis. Nat. Methods 9, 676–682. 10.1038/nmeth.2019. 22743772 PMC3855844

[R83] Schmidt G, Sehr P, Wilm M, Selzer J, Mann M, and Aktories K (1997). Gln 63 of Rho is deamidated by Escherichia coli cytotoxic necrotizing factor-1. Nature 387, 725–729. 10.1038/42735. 9192900

[R84] Schrage R, Schmitz A-L, Gaffal E, Annala S, Kehraus S, Wenzel D, Büllesbach KM, Bald T, Inoue A, Shinjo Y, (2015). The experimental power of FR900359 to study Gq-regulated biological processes. Nat. Commun 6, 10156. 10.1038/ncomms10156. 26658454 PMC4682109

[R85] Seifert R, and Wenzel-Seifert K (2002). Constitutive activity of G-protein-coupled receptors: cause of disease and common property of wild-type receptors. Naunyn-Schmiedebergs Arch. Pharmacol 366, 381–416. 10.1007/s00210-002-0588-0. 12382069

[R86] Sosinsky GE, Crum J, Jones YZ, Lanman J, Smarr B, Terada M, Martone ME, Deerinck TJ, Johnson JE, and Ellisman MH (2008). The combination of chemical fixation procedures with high pressure freezing and freeze substitution preserves highly labile tissue ultrastructure for electron tomography applications. J. Struct. Biol 161, 359–371. 10.1016/j.jsb.2007.09.002. 17962040 PMC2459253

[R87] Sun Z, Wang B, Chen C, Li C, and Zhang Y (2021). 5-HT6R null mutation induces synaptic and cognitive defects. Aging Cell 20, e13369. 10.1111/acel.13369. 33960602 PMC8208783

[R88] Tai Y, Gallo NB, Wang M, Yu J-R, and Van Aelst LV (2019). Axo-axonic innervation of neocortical pyramidal neurons by GABAergic chandelier cells requires AnkyrinG-associated L1CAM. Neuron 102, 358–372.e9. 10.1016/j.neuron.2019.02.009. 30846310 PMC6525570

[R89] Takasaki J, Saito T, Taniguchi M, Kawasaki T, Moritani Y, Hayashi K, and Kobori M (2004). A novel Gáq/11-selective inhibitor. J. Biol. Chem 279, 47438–47445. 10.1074/jbc.M408846200. 15339913

[R90] Tereshko L, Gao Y, Cary BA, Turrigiano GG, and SenGupta P (2021). Ciliary neuropeptidergic signaling dynamically regulates excitatory synapses in postnatal neocortical pyramidal neurons. eLife 10, e65427. 10.7554/eLife.65427. 33650969 PMC7952091

[R91] Delgado TC, Petralia RS, Freeman DW, Sedlacek M, Wang Y-X, Brenowitz SD, Sheu S-H, Gu JW, Kapogiannis D, Mattson MP, and Yao PJ (2019). Comparing 3D ultrastructure of presynaptic and postsynaptic mitochondria. Biol. Open 8, bio044834. 10.1242/bio.044834. 31362947 PMC6737966

[R92] Thompson CL, Ng L, Menon V, Martinez S, Lee C-K, Glattfelder K, Sunkin SM, Henry A, Lau C, Dang C, (2014). A high-resolution spatiotemporal atlas of gene expression of the developing mouse brain. Neuron 83, 309–323. 10.1016/j.neuron.2014.05.033. 24952961 PMC4319559

[R93] Ting JT, Lee BR, Chong P, Soler-Llavina G, Cobbs C, Koch C, Zeng H, and Lein E (2018). Preparation of acute brain slices using an optimized N-methyl-D-glucamine protective recovery method. J. Vis. Exp 132, e53825. 10.3791/53825. PMC5931343 29553547

[R94] Tu H-Q, Li S, Xu Y-L, Zhang Y-C, Jian X-X, Song G-P, Wu M, Song Z-Q, Hu H-B, Li P-Y, (2022). Rhythmic cilium in SCN neuron is a gatekeeper for the intrinsic circadian clock. Preprint at bioRxiv. 10.1101/2022.01.26.477948.

[R95] Upton N, Chuang TT, Hunter AJ, and Virley DJ (2008). 5-HT6 receptor antagonists as novel cognitive enhancing agents for Alzheimer’s disease. Neurotherapeutics 5, 458–469. 10.1016/j.nurt.2008.05.008. 18625457 PMC5084247

[R96] Viita T, Kyheröinen S, Prajapati B, Virtanen J, Frilander MJ, Varjosalo M, and Vartiainen MK (2019). Nuclear actin interactome analysis links actin to KAT14 histone acetyl transferase and mRNA splicing. J. Cell Sci 132, jcs226852. 10.1242/jcs.226852. 30890647 PMC6503952

[R97] Wan J, Peng W, Li X, Qian T, Song K, Zeng J, Deng F, Hao S, Feng J, Zhang P, (2021). A genetically encoded sensor for measuring serotonin dynamics. Nat. Neurosci 24, 746–752. 10.1038/s41593-021-00823-7. 33821000 PMC8544647

[R98] Wan OW, Shin E, Mattsson B, Caudal D, Svenningsson P, and Björklund A (2016). α-synuclein induced toxicity in brain stem serotonin neurons mediated by an AAV vector driven by the tryptophan hydroxylase promoter. Sci. Rep 6, 26285. 10.1038/srep26285. 27211987 PMC4876322

[R99] Williams SL, Lutz S, Charlie NK, Vettel C, Ailion M, Coco C, Tesmer JJ, Jorgensen EM, Wieland T, and Miller KG (2007). Trio’s Rho-specific GEF domain is the missing Gαq effector in *C. elegans*. Genes Dev 21, 2731–2746. 10.1101/gad.1592007. 17942708 PMC2045128

[R100] Wong PT, Roberts EW, Tang S, Mukherjee J, Cannon J, Nip AJ, Corbin K, Krummel MF, and Choi SK (2017). Control of an unusual photo-Claisen rearrangement in coumarin caged tamoxifen through an extended spacer. ACS Chem. Biol 12, 1001–1010. 10.1021/acschembio.6b00999. 28191924 PMC5404426

[R101] Wu Y, Whiteus C, Xu CS, Hayworth KJ, Weinberg RJ, Hess HF, and De Camilli P (2017). Contacts between the endoplasmic reticulum and other membranes in neurons. Proc. Natl. Acad. Sci. USA 114, E4859–E4867. 10.1073/pnas.1701078114. 28559323 PMC5474793

[R102] Xu CS, Pang S, Hayworth KJ, and Hess HF (2020). Transforming FIB-SEM systems for large-volume connectomics and Cell Biology. In Volume Microscopy, Wacker I, Hummel E, Burgold S, and Schröder R, eds. (Springer), pp. 221–243. 10.1007/978-1-0716-0691-9_12.

[R103] Yang J, Liu X, Yue G, Adamian M, Bulgakov O, and Li T (2002). Rootletin, a novel coiled-coil protein, is a structural component of the ciliary rootlet. J. Cell Biol 159, 431–440. 10.1083/jcb.200207153. 12427867 PMC2173070

[R104] Yu X, Zhang Q, Zhao Y, Schwarz BJ, Stallone JN, Heaps CL, and Han G (2017). Activation of G protein-coupled estrogen receptor 1 induces coronary artery relaxation via Epac/Rap1-mediated inhibition of RhoA/Rho kinase pathway in parallel with PKA. PLoS One 12, e0173085. 10.1371/journal.pone.0173085. 28278256 PMC5344336

[R105] Zhao K, Wang W, Rando OJ, Xue Y, Swiderek K, Kuo A, and Crabtree GR (1998). Rapid and phosphoinositol-dependent binding of the SWI/SNF-like BAF complex to chromatin after T lymphocyte receptor signaling. Cell 95, 625–636. 10.1016/S0092-8674(00)81633-5. 9845365

[R106] Zheng Q, Ayala AX, Chung I, Weigel AV, Ranjan A, Falco N, Grimm JB, Tkachuk AN, Wu C, Lippincott-Schwartz J, (2019). Rational design of fluorogenic and spontaneously blinking labels for super-resolution imaging. ACS Cent. Sci 5, 1602–1613. 10.1021/acscentsci.9b00676. 31572787 PMC6764213

[R107] Zieba BJ, Artamonov MV, Jin L, Momotani K, Ho R, Franke AS, Neppl RL, Stevenson AS, Khromov AS, Chrzanowska-Wodnicka M, (2011). The cAMP-responsive Rap1 guanine nucleotide exchange factor, epac, induces smooth muscle relaxation by down-regulation of RhoA activity. J. Biol. Chem 286, 16681–16692. 10.1074/jbc.M110.205062. 21454546 PMC3089510

