# Supplementary material for: A serotonergic axon-cilium synapse drives nuclear signaling to alter chromatin accessibility
Source: Cell. Author manuscript; Available in PMC 2022 Dec 24. (PMC9789380; doi:10.1016/j.cell.2022.07.026)
Supplement: Methods S1 [file NIHMS1851533-supplement-Methods_S1.pdf]

1 M E T D T L L L W V L L L W V P G S T G D T S L Y K K V G T T G V P E P G P T A N S T P A W G A G P 50

51 P S A P G G S G W V A A A L C V V I A L T A A A N S L L I A L I C T Q P A L R N T S N F F L V S L F 100

101 T S D L M V G L V V M P P A M L N A L Y G R W V L A R G L C L L W T A F D V M C C S A S I L N L C L 150

151 I S L D R Y L L I L S P L R Y K L R M T P L R A L A L V L G A W S L A A L A S F L P L L L G W H E L 200

201 G H A R P P V P G Q C R L L A S L P F V L V A S G L T F F L P S G A I C F T Y C R I L L A A R K Q A 250

251 V Q V A S L T T G M A S Q R G P D A V A A P P G G T E R R P N G L G P E R S A G P G G A E A E P L P 300

301 T Q L N G A P G E P A P A G P R D T D A L D L E E G G N V Y I K A D K Q K N G I K A N F H I R H N I 350

351 E D G G V Q L A Y H Y Q Q N T P I G D G P V L L P D N H Y L S V Q S K L S K D P N E K R D H M V L L 400

401 E F V T A A G I T L G M D E L Y K G G T G G S M V R K G E E L F T G V V P I L V E L D G D V N G H K 450

451 F S V S G E G E G D A T E G K L T L K F I C T T G K L P V P W P T L V T T L T Y G V Q C F S R Y P D 500

501 H M K Q H D F F K S A M P E G Y I Q E R T I F F K D D G N Y K T R A E V K F E G D T L V N R I E L K 550

551 G I D F K E D G N I L G H K L E Y N T G A A A R W H S R K A L K A S L S L G I L L G M F F V T W L P 600

601 F F V A N I V Q A V C D C I S P G L F D V L T W L G Y C N S T M N P I I Y P L F M R D F K R A L G R 650

651 F L P C P R C P R E R Q A S L A S P S L R T S H S G P R P G L S L Q Q V L P L P L P P D S D S D S D 700

701 A G S G G S S G L R L T A Q L L L P G E A T Q D P P L P T R A A A A V N F F N I D P A E P E L R P H 750

751 P L G I P T I R L A P L S L P P P * 800

Methods S1. The amino acid sequence of the HTR6-PM sensor. Related to STAR Methods. The numbering starts from the IgK leader sequence. Insertion sites Q263ICL3 and H5766.26 are indicated by black arrowheads. Asterisk indicates the stop codon.
