# Supplementary material for: A serotonergic axon-cilium synapse drives nuclear signaling to alter chromatin accessibility
Source: Cell. Author manuscript; Available in PMC 2022 Dec 24. (PMC9789380; doi:10.1016/j.cell.2022.07.026)
Supplement: 3 [file NIHMS1851533-supplement-3.pdf]

CellPress
OPEN ACCESS

Supplemental figures

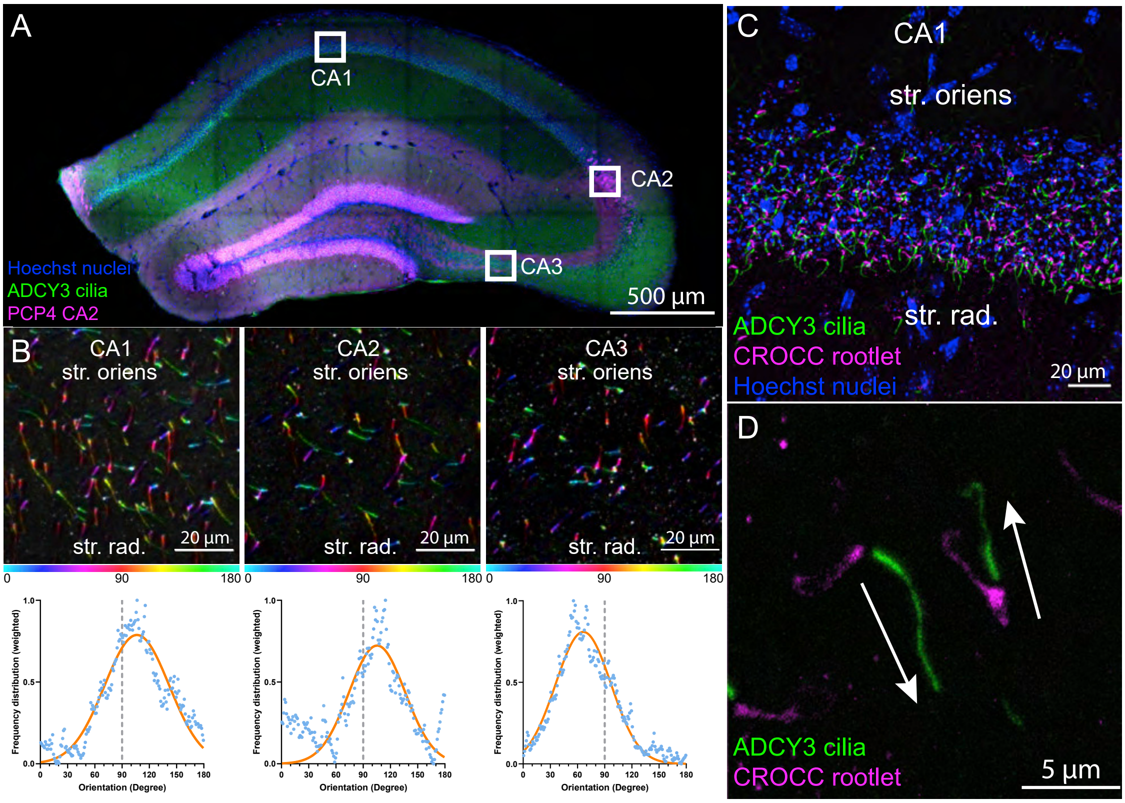

Figure S1. Adult hippocampal pyramidal neuronal cilia are oriented, related to Figure 1

(A) Hippocampal coronal 200 μm-thick section maximum intensity projection (MIP). Green: cilia (ADCY3), red: CA2 (PCP4), and blue: nuclei (Hoechst 33342).

(B) Orientation (structure tensor) analyses of cilia voxels in CA1, CA2, and CA3 (200 μm MIP), showing cilia oriented along the basal-apical axis (stratum oriens → stratum radiatum). The images are rotated such that the basal-apical axes are at a 90° angle. Top panel: color survey of cilia voxels encoded by orientation (hue), coherence (saturation), and fluorescence intensity (brightness). Bottom panel: normalized weighted frequency distribution with basal-apical axes at 90°, showing original data (blue) and fitted Gaussian curves (orange). Mean values of the Gaussian distributions are 106°, 106°, and 66° for CA1, CA2, and CA3, respectively. The tail distributions of CA2 fit less well than for CA1 and CA3, indicating more heterogeneity in CA2 cilia vectors.

(C) Labeling of ciliary base. CA1 cilia (green) and Rootletin (magenta; CROCC, ciliary rootlet); nuclei (blue, Hoechst 33342); 50 μm MIP.

(D) Two cilia oriented at 180° in (C) are magnified. 5 μm MIP. Data were from 3-month-old male C57BL/6J mice.

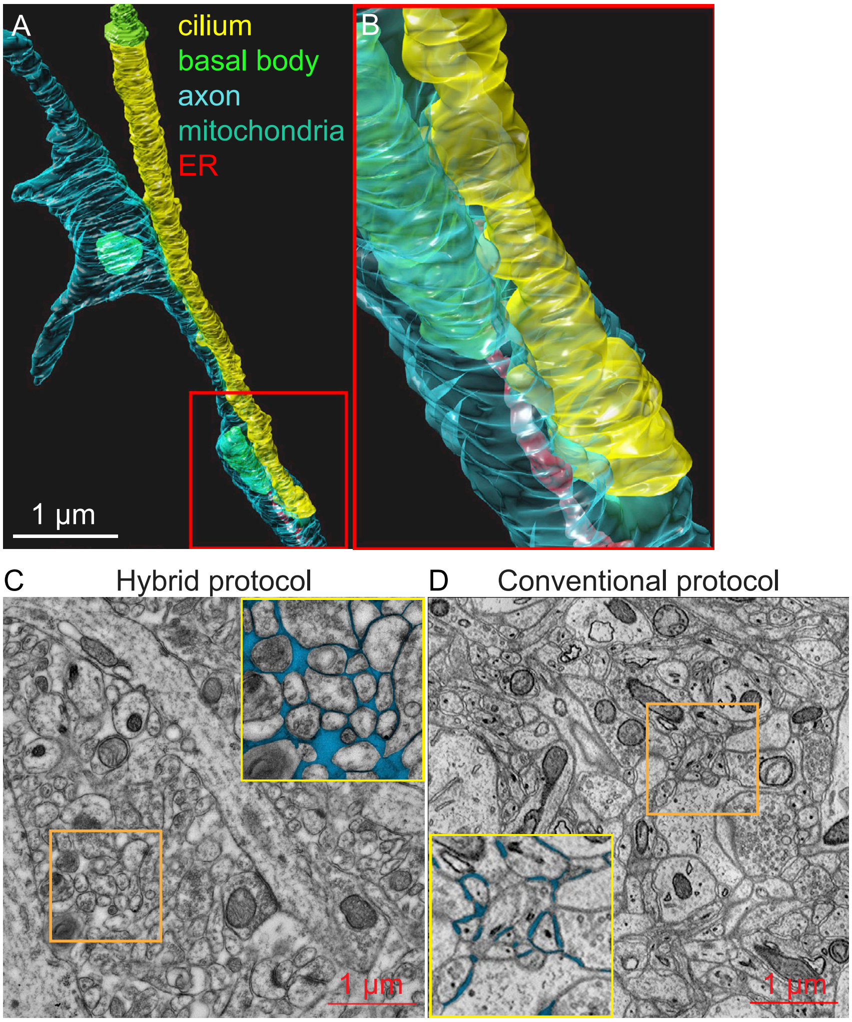

Figure S2. FIB-SEM reconstruction of a P14 (juvenile) CA1 pyramidal neuron and the hybrid protocol, related to Figure 1

(A and B) The neuronal cilium fasciculates with an axonal process. Juvenile axo-ciliary synapses are similar to those in adult pyramidal neuronal cilia. Yellow: cilium, cyan: axon, bright green: basal body, red: axonal endoplasmic reticulum, and green: axonal mitochondria.

(C and D) In contrast to conventional glutaraldehyde perfusion protocols (Kasthuri et al., 2015; D, orange box magnified in the inset with yellow border), the hybrid protocol introduced here preserves the extracellular space (C, orange box magnified in the inset with yellow border). Note the rounded morphology of neuronal processes and significantly greater extracellular space (blue).

CellPress
OPEN ACCESS

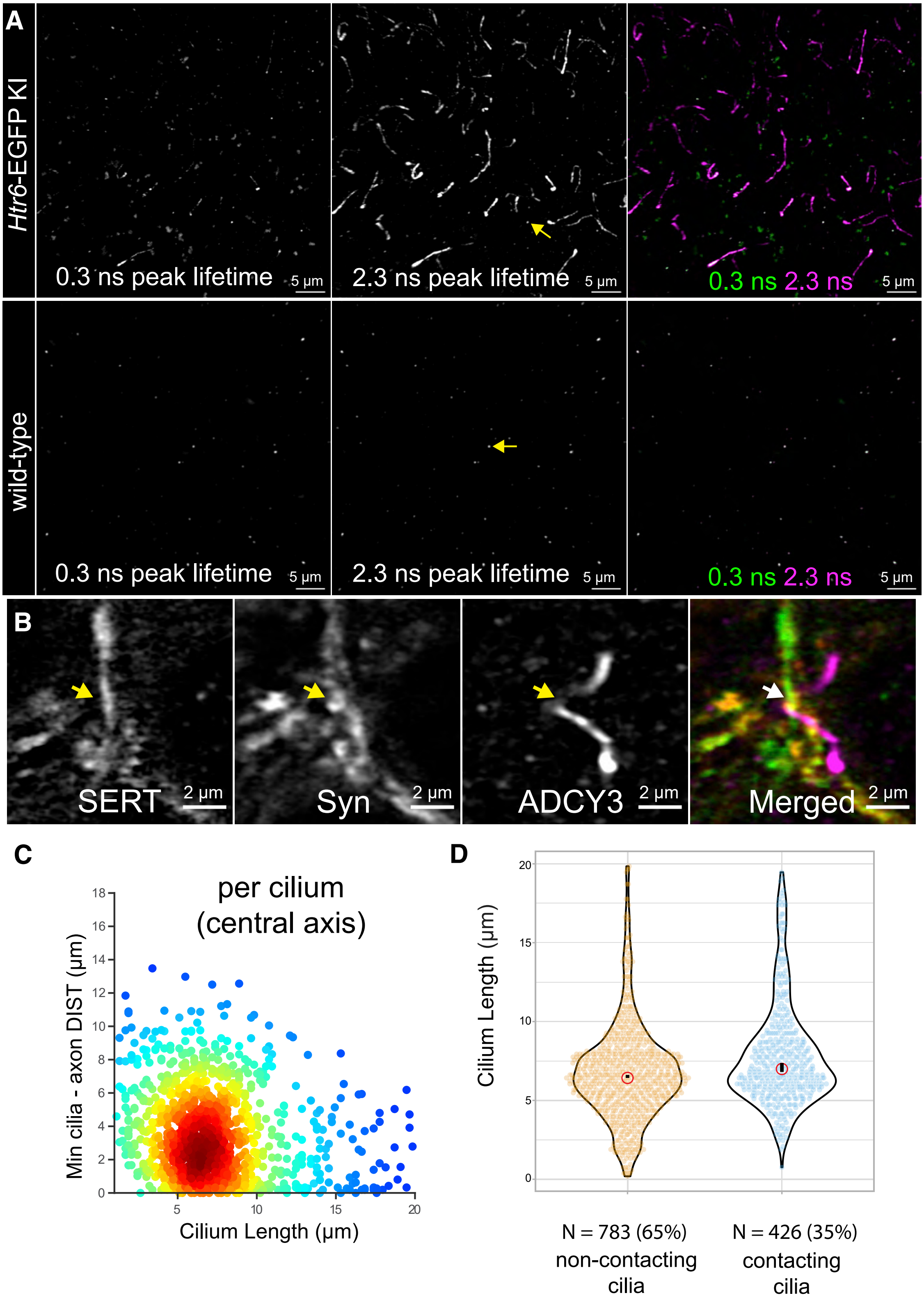

(legend on next page)

Figure S3. Neuronal primary cilia contact serotonergic axons, related to Figure 2

(A) Fluorescence lifetime separation of GFP signals (amplified by anti-GFP antibody and CF633 dye) from autofluorescence background in the far-red channel. Autofluorescence lifetimes peak around 0.3 ns (left panels) and CF633 fluorescence lifetimes around 2.3 ns (middle panels). Notice that the small puncta with 2.3 ns lifetimes are also present in non-EGFP wild-type controls, suggesting that these are from non-specific antibody labeling. 20 μm MIP.

(B) Magnified from Figure 2C: a cilium contacting a serotonergic synaptophysin-labeled axonal varicosity. The serotonergic axon (SERT), synaptophysin (Syn), and cilia (ADCY3) are colored in green, yellow, and magenta in the merged panel, respectively.

(C) Density plot showing the relationship between cilia length and shortest distance to a serotonergic axon per cilium (central axis). Note the lack of a linear correlation (Pearson correlation coefficient $r = -0.19$) and the skewed distribution toward shorter distances.

(D) Violin plots showing the distribution of ciliary lengths of serotonergic axon-contacting and non-contacting cilia. The difference in the median is statistically significant ($p < 0.0001$, two-tailed Mann-Whitney U test). Red circle: median; black bar: 95% confidence interval of the median. The difference in the shapes of the violin plots and the length of the 95% bars reflect the slightly greater variance of contacting cilia (standard deviation: 3.3 μm for contacting cilia, versus 3.0 μm for non-contacting cilia; two-group distribution comparison p value with permutation test <0.0001).

CellPress
OPEN ACCESS

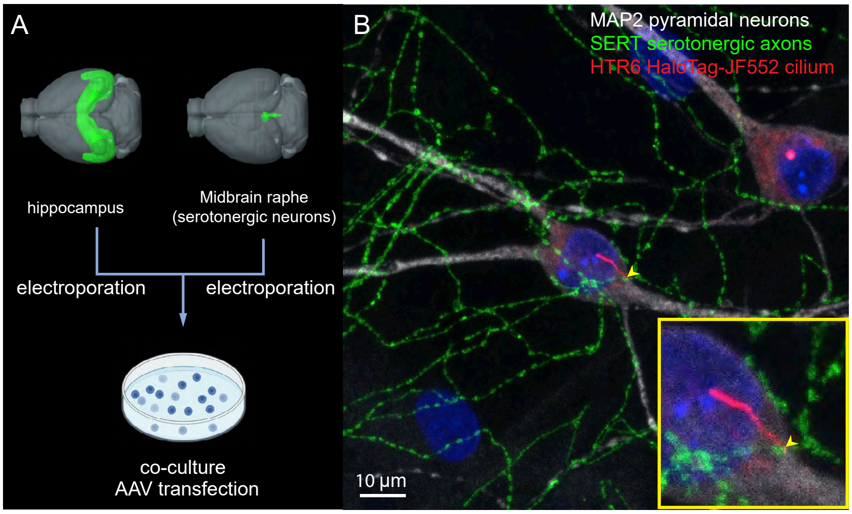

Figure S4. Serotonergic axo-ciliary synapse *in vitro*, related to Figure 4

(A) Overall workflow (upper images, Allen Brain Explorer; Lau et al., 2008). Hippocampal and raphe neurons were dissociated from the hippocampus and midbrain, respectively. In some experiments, constructs were electroporated separately before co-plating in the same well (STAR Methods).

(B) Serotonergic axo-ciliary synapses *in vitro*. On average (1 million cell total, 300,000/cm^2 density, 1:1 hippocampal and midbrain cell ratio), there are 5–10 serotonergic neurons and ~5 axo-ciliary synapses per well.

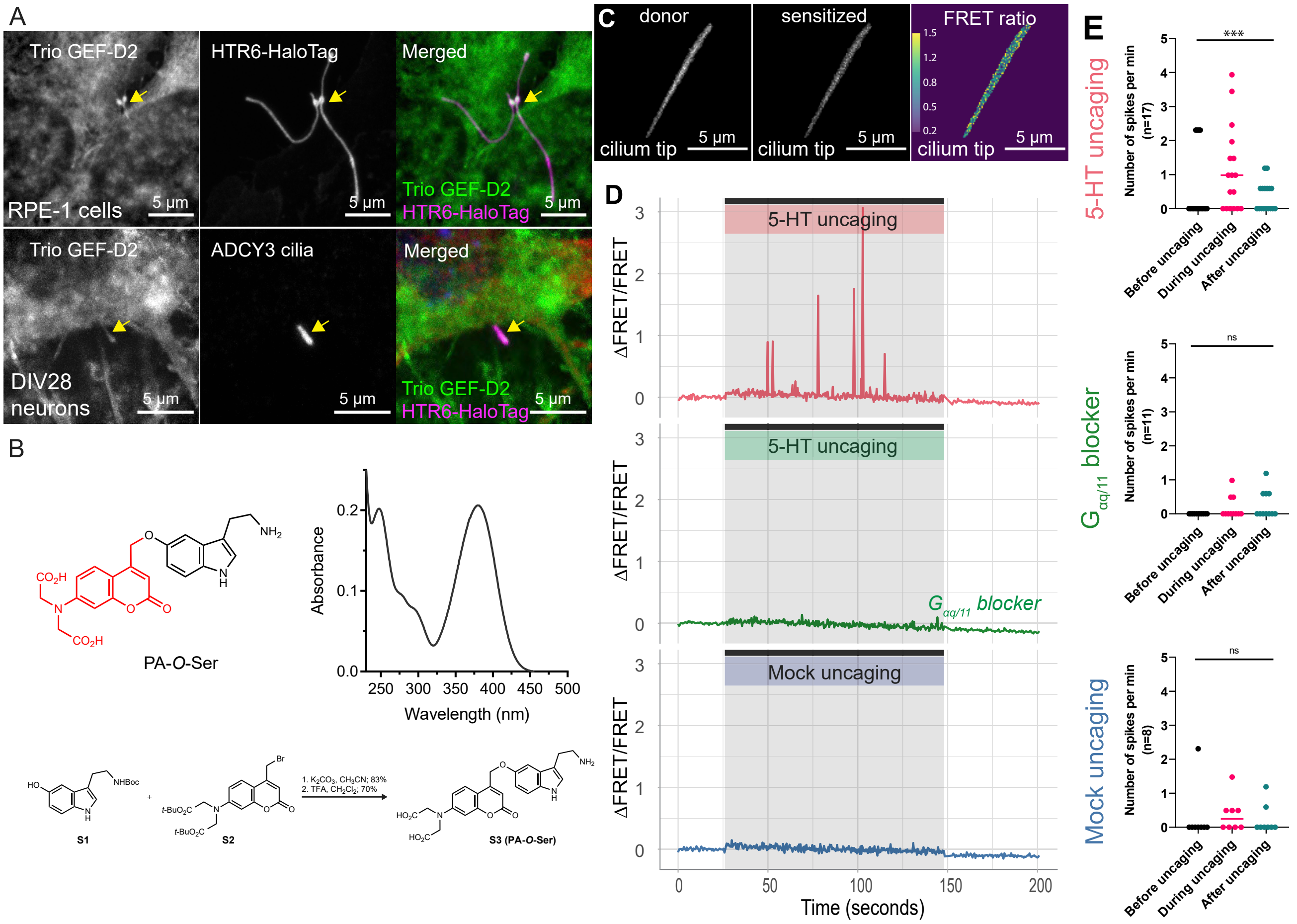

Figure S5. Ciliary $G_{\alpha q}$-Trio-RhoA signaling in RPE-1 cells, related to Figure 5

(A) Trio is present in HTR6-cilia of RPE-1 cells and WT-cultured hippocampal neuronal cilia. Top panel: RPE-1 cells stably expressing the Tet-inducible HTR6-HaloTag. HaloTag was labeled with Janelia Fluor 552 (magenta in the merged panel), fixed, and stained with an antibody against the Trio GEF-D2 domain (green, merged panel). Lower panel: Trio is present in WT-cultured hippocampal neuronal cilia. DIV28 cultured rat hippocampal neurons were fixed and immunostained with anti-ADCY3 antibody (neuronal cilia marker, magenta; merged panel), anti-MAP2 antibody (neuronal marker, red; merged panel), Hoechst 33342 (nucleus, blue; merged panel), and anti-Trio GEF-D2 antibody (green, merged panel). The Trio GEF-D2 signal in both cases was amplified with the Alexa 488 tyramide signal amplification system (Thermo Fisher Scientific). Images were processed with the Subtract Background (50 pixels with sliding paraboloid) algorithm in ImageJ/Fiji to enhance contrast for qualitative demonstrations.

(B) Properties of photo-activatable (“caged”) serotonin (PA-Ser). Top left: chemical structure of PA-Ser. Top right: absolute absorption spectrum of a solution of PA-Ser (10 μM) in PBS. This molecule displayed an absorption maximum of 380 nm with an extinction coefficient (ε) of 21,100 $M^{-1}cm^{-1}$; the relatively broad absorption spectrum gives substantial absorption at 405 nm (ε = 12,100 $M^{-1}cm^{-1}$). Upon photolysis, PA-Ser releases ~10% of serotonin along with other major photoproducts generated primarily via a photo-Claisen pathway (Wong et al., 2017). Lower panel: synthesis of PA-Ser through alkylation of Boc-protected serotonin (S1) with {7-[bis(carboxymethyl)amino] coumarin-4-yl} methyl (BCMACM) bromide (S2). This coumarin-based BCMACM photolabile group exhibits high aqueous solubility and relatively large one- and two-photon activation cross-sections (Hagen et al., 2008).

(C–E) Serotonin stimulation of ciliary HTR6 activates RhoA. RPE-1 cells stably expressing a Tet-inducible HTR6-RhoA FRET-based sensor. (C) Donor emission (sGFP2), sensitized emission (mScarlet-I), and FRET ratio calculated by dividing sensitized emission by donor emission of a single cilium. Local serotonin uncaging at 0.5 Hz results in RhoA activity spikes. (D) Top panel shows a sample trace, quantified in (E) top panel, p value = 0.04. The effect is largely attenuated by pretreating samples with the $G_{\alpha q/11}$ blocker, YM-254890 (1 μM), (D) middle panel is a sample trace, quantified in (E) middle panel, p value = 0.12. Mock uncaging had minimal effect on the RhoA FRET ratio. (D) Lower panel is a sample trace, quantified in (E), lower panel, p value = 0.52. For (E), the spikes are defined as ΔF/F greater than or equal to 0.52 (the mean ± 3 SD in the 5-HT uncaging measurements). Horizontal lines represent the median values. Statistical tests comparing before, during, and after uncaging used the Friedman test (non-parametric).

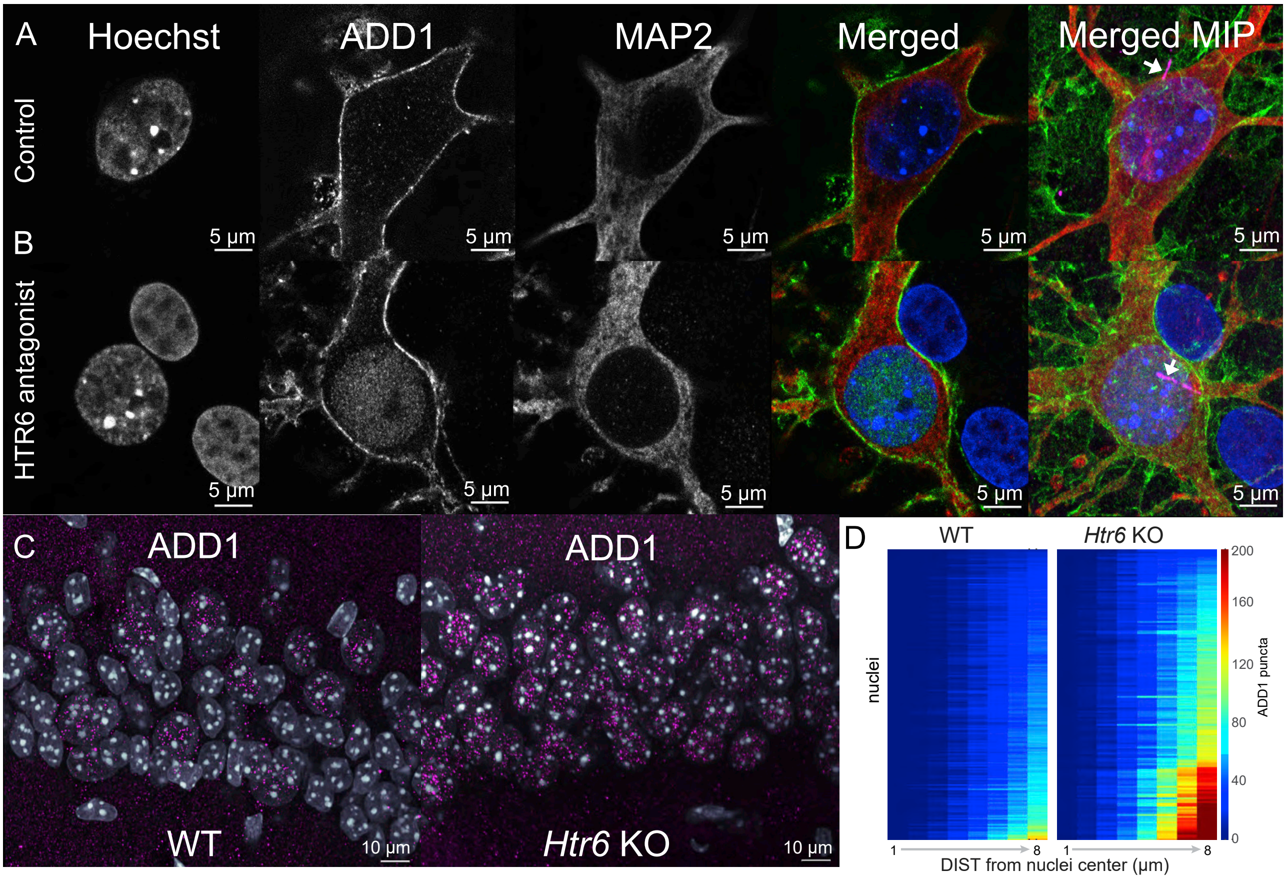

Figure S6. Modulation of the 5-HTR6 signaling axis alters adducin localization, related to Figure 6

(A and B) DIV28 hippocampal neurons were treated either with 0.01% DMSO control (A) or 100 nM SB-742457 (B) for 20 min. In DMSO-treated neurons, adducin is primarily at the plasma membrane. In contrast, in some SB-742457-treated neurons, there is significant nuclear labeling. Pyramidal neurons are identified by MAP2 labeling in both cases. Color scheme in merged panels: blue: Hoechst 33342, green: adducin, and red: MAP2. Cilia are colored in magenta (ADCY3 staining, arrow) in the merged MIP of the entire neuron, while other panels are single optical sections through the middle of the nucleus.

(C) *Htr6* KO mice exhibit increased numbers of pyramidal neurons with nuclear adducin (ADD1) puncta. Data were from 4-month-old male C57BL/6 mice.

(D) Heatmap of a number of ADD1 nuclear puncta (color coded) using the center of the nucleus (represented in rows) with increasing distance from 1 to 8 µm (represented in columns). Punctal density is clearly increased in KO cells. Mean difference by estimation statistics between WT and KO cells within 5-µm radius from the center of nuclei = 25.5, 95% CI = 20.4–47.6, permutation test p value = 0, two-tailed Mann-Whitney test p value < 0.0001.

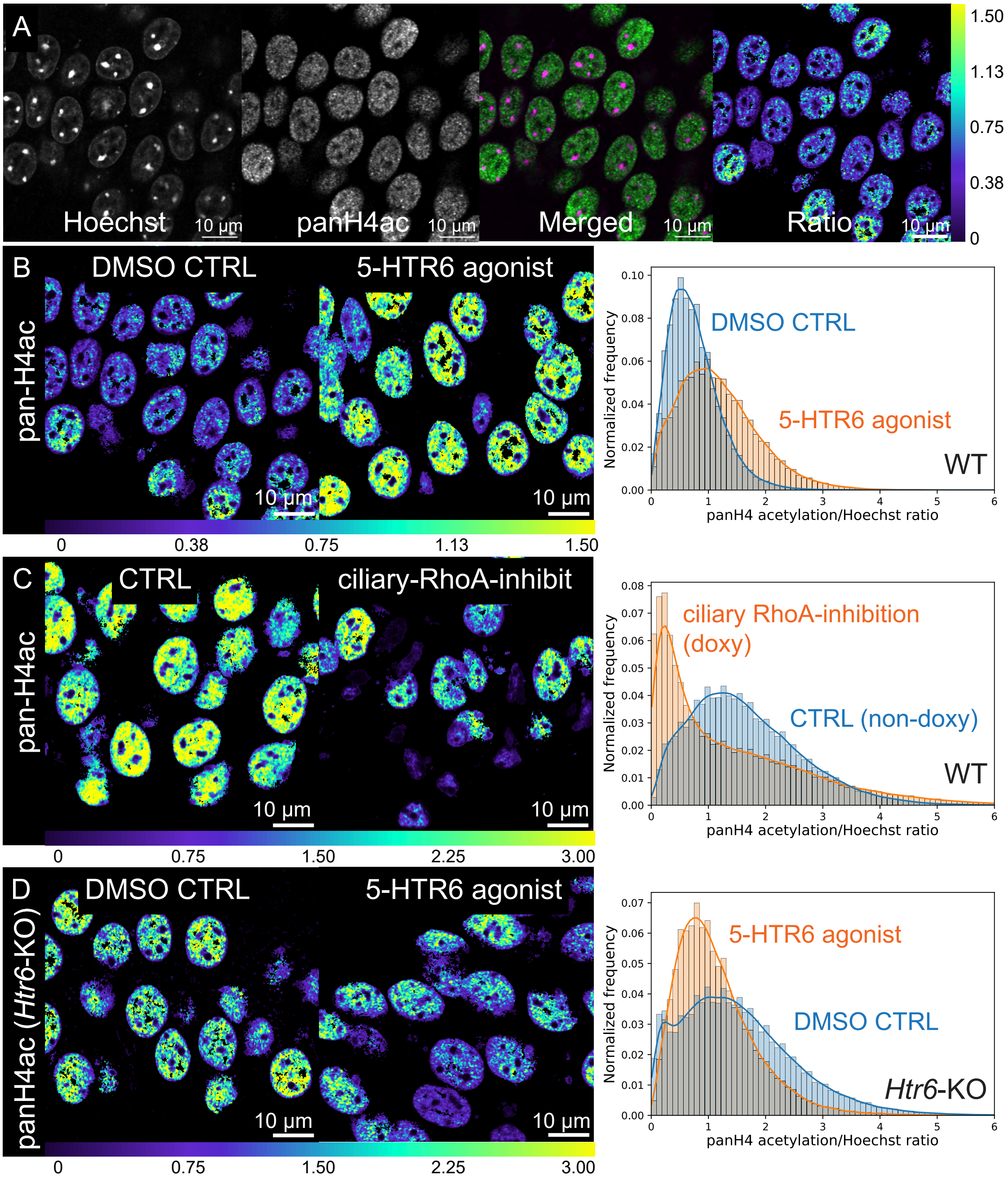

Figure S7. 5-HTR6 signaling modulates histone H4 acetylation, related to Figure 6

(A) Ratiometric measurements of pan-H4 acetylation in fixed mouse brain sections. Monoclonal antibodies to acetylated H4 were used to detect histone H4 lysine acetylation (pan-H4ac, green in the merged panel). The fluorescent intensity is further divided by the Hoechst intensity levels (magenta in the merged panel) to obtain the ratio (downsampled in x-y, STAR Methods) in the rightmost panel. Shown are representative single Airyscan optical sections.

(B–D) The pan-H4ac/Hoechst ratio is significantly increased with 5-HTR6 agonist application (B, 71% increase in mode; 0.56 versus 0.96), decreased in the ciliary RhoA inhibition (C, 80% decrease in mode; 1.27 versus 0.25). 5-HTR6 agonist stimulation did not increase the pan-H4ac/Hoechst ratio in *Htr6* KO mice (D, 30% decrease in mode; 1.05 versus 0.74). Left and middle panels: single optical sections. Right panel: histograms with kernel density estimates from entire stacks. Data in (A) and (B) were from 3- to 3.5-month-old and in (C) and (D) were from 4-month-old male C57BL/6J mice.
